# Perceived parenting and identification with all humanity: Insights from England and Germany

**DOI:** 10.3389/fpsyg.2022.924562

**Published:** 2022-08-03

**Authors:** Minne Luise Hagel, Friedemann Trutzenberg, Michael Eid

**Affiliations:** Department of Education and Psychology, Freie Universität Berlin, Berlin, Germany

**Keywords:** identification with all humanity, cosmopolitanism, parenting behavior, attachment, structural equation modeling

## Abstract

In the past decade, identification with all humanity (IWAH) has been found to predict several positive behavioral outcomes like volunteering, a willingness to contribute to humanitarian relief, and cooperative health behavior during the COVID-19 pandemic. However, to this day, little is known about how individual differences in IWAH emerge. Therefore, the present study aimed to explore whether there is a relationship between individuals’ upbringing and their IWAH. For this purpose, data on IWAH, remembered parenting behavior (RPB), and remembered parental attachment assessed by 3056 individuals (1517 from Germany and 1539 from England) were analyzed. Structural equation models were used to (A) analyze the correlations between RPB, attachment, and IWAH and to (B) test whether single facets of RPB and attachment could significantly predict IWAH when controlling for the other facets in a latent regression analysis. The facets of positive RPB correlated significantly positively with the two facets of IWAH (global self-definition and global self-investment) and explained between 4.1 and 7% of their variance. Surprisingly, in the English sample, two facets of negative RPB also correlated significantly positively with IWAH. The explained variance in IWAH being significant but small, it is argued that parents’ attitudes or behavior specifically related to IWAH could have a greater impact on IWAH than more unspecific parenting behavior. For instance, we discovered that the extent to which participants perceived their parents as global citizens explained about one third of the variance in their own identification as global citizens. Fostering IWAH could constitute an effective approach to tackle important global challenges. Therefore, more research is needed to test the generalizability of the results and to further analyze the roots of people’s IWAH.

## Introduction

Being asked where he came from, the ancient Greek philosopher Diogenes of Sinope (c. 412 B.C.) stated: “I am a citizen of the world (kosmopolitês)” (Caraus, 2021, p. 3). Due to his declaration, Diogenes of Sinope is regarded as the first person coining the term *cosmopolitanism* (Leung et al., 2015; Caraus, 2021). Since then, the term has increasingly been used to describe different positions supporting the notion of a literal or metaphorical world citizenship (Kleingeld, 2013). From a cosmopolitan perspective, all human beings belong to one community, sometimes called *the cosmopolis* (Pierik and Werner, 2010). Cosmopolitanism can also refer to a moral ideal which highlights the equal moral worth of all people and entails obligations for all people to protect this worth (Pierik and Werner, 2010).

In today’s globalized world, cosmopolitan ideas are repeatedly brought up by famous politicians. In June 2008, during a speech in Berlin, Barack Obama stepped into Diogenes’ footsteps by declaring: “I speak (…) as a fellow citizen of the world” (Selzer, 2010). A quite oppositional position was voiced by former British Prime Minister Theresa May. In a speech following the Brexit referendum in 2016, she said: “(…) if you believe you are a citizen of the world, you are a citizen of nowhere. You don’t understand what the very word citizenship means” (Bearak, 2016). But not only politicians show interest in the idea of global citizenship. Cosmopolitanism is also lively discussed by researchers from various disciplines. Depending on their field, they offer different perspectives on the subject (Sevincer et al., 2017). In philosophy, political science and sociology, discussions on cosmopolitanism usually focus on global ethics and the difficulties of their implementation (van Hooft, 2014; Sevincer et al., 2017; Nussbaum, 2019).

In psychology, cosmopolitanism and global citizenship are mainly studied on an individual level (Reysen and Katzarska-Miller, 2018). The idea of an individual value orientation that consists in considering mankind as one’s primary reference group was already discussed over 60 years ago by Sampson and Smith (1957) who named such an orientation world-mindedness. Early studies on world-mindedness included the investigation of its relationship to perceived nuclear threat and anti-nuclear activism during the Cold War (Der-Karabetian, 1992). References to global citizenship can be found in publications in many other disciplines too, like agriculture, geography, economics, arts, computer science and medicine (Sevincer et al., 2017; Reysen and Katzarska-Miller, 2018). Some topics are also being discussed through a cosmopolitan lens by researchers across disciplinary boundaries. Two particularly important examples are the climate change on our shared planet (Der-Karabetian et al., 1996, 2014; Pierik and Werner, 2010; Page, 2011; Jaspal et al., 2014; Der-Karabetian and Alfaro, 2015; Lee et al., 2015; Leung et al., 2015; Reese, 2016; Loy and Spence, 2020; Pong, 2020) and global migration (Pierik and Werner, 2010; Patterson and Choi, 2018; Sparkman and Hamer, 2020). Considering the importance of these and other global challenges, calls for interdisciplinary work on cosmopolitanism have been rising over the past few years (Cameron, 2018; Reysen and Katzarska-Miller, 2018).

Now, what makes the idea of a global community relevant for individuals’ experience and behavior? Studying the origins of altruism, Monroe (1996) interviewed people who had shown exceptional altruistic behavior in the past. For instance, she asked people who had rescued Jews during the Holocaust what had led them to risk their own safety to help others. Hearing her answers, Monroe was “struck by the similarity of expressions used” (p. 205), because “the idea of being welded together, of belonging to one human family, surfaced over and over again” (p. 205). By publishing parts of her interviews, she impressively showed that the notion of a shared human family could animate people to help and protect others.

### Correlates of identification with all humanity

Inspired by Monroe’s reports, McFarland et al. (2012) started exploring the rescuers’ shared motive. They wanted to find out why some people identified with the “human family” more than others and what consequences this identification might have. To facilitate their studies, they introduced a new psychological construct describing individual differences in “viewing all humanity as family” and named it “identification with all humanity (IWAH)” (McFarland et al., 2012). Later, McFarland et al. (2013) also defined IWAH as “a deep caring for all human beings regardless of their race, religion, or nationality” (p. 194).

Variables which have been found to correlate positively with IWAH include perspective taking and empathetic concern (about *r* = 0.30, respectively *r* = 0.50; McFarland et al., 2012), openness to experience (about *r* = 0.29 to *r* = 0.39; McFarland et al., 2019), horizontal collectivism (about *r* = 0.30 to *r* = 0.40; McFarland et al., 2012), agreeableness (about *r* = 0.20 to *r* = 0.33; ibid.), and logical reasoning (*r* = 0.21; ibid.). Variables that have been found to correlate negatively with IWAH include generalized prejudice (ethnocentrism; Altemeyer, 1996), right-wing authoritarianism (RWA; ibid.), and social dominance orientation (SDO; Sidanius and Pratto, 1999), with correlations between IWAH and these three variables usually ranging from *r* = −0.30 to *r* = −0.60 (McFarland et al., 2019). Other negative correlations have been observed between IWAH and the support for building a wall on the American-Mexican border (*r* = −0.36; ibid.), the need for social approval (up to *r* = −0.34; ibid.), and psychopathy (*r* = −0.20 to *r* = −0.46; ibid.). For a more extensive overview of correlates of IWAH, see McFarland et al. (2019).

### Parenting and identification with all humanity

Coming back to McFarland’s et al. (2012) original intentions to study IWAH: They wanted to find out why some people identify with the “human family” more than others and what consequences this identification might have. Using regression and structural equation models (SEM), a few possible answers to these questions have already been found. IWAH has been found to predict the desire for global knowledge, a willingness to contribute to humanitarian relief, forgiving former enemies of their war crimes (Hamer et al., 2017, 2018), volunteering (Stürmer et al., 2016; Faulkner, 2018), and cooperative health behavior during the COVID-19 pandemic (Barragan et al., 2021; Marchlewska et al., 2022; Sparkman et al., 2022). Examples for variables that have been found to predict IWAH itself are global awareness, openness to experience, empathy, universalism (Hamer et al., 2019), and multicultural experiences (Sparkman and Hamer, 2020). In the present study, we also focus on IWAH’s roots by investigating possible origins in childhood.

The way parents raise their children can be described using several different concepts. One popular way to do so is using parenting styles. A parenting style can be defined as a “constellation of attitudes toward the child that are communicated to the child and that, taken together, create an emotional climate in which the parent’s behaviors are expressed” (Darling and Steinberg, 1993, p. 488). Parenting styles are relatively stable over different situations and points in time (Cierpka, 2008). They can be subdivided into three more specific characteristics: “the values and goals parents have in socializing their children, the parenting practices they employ, and the attitudes they express toward their children” (Darling and Steinberg, 1993, p. 492). In the past few decades, the term “parenting behavior” has often been used as a synonym for the term “parenting style” (Cierpka, 2008; Rueger et al., 2011). According to Feldman (2012), the term “parenting behavior” has a slight focus on objectively observable actions, which can form a behavioral style when shown repeatedly (p. 536). In the present study, the term “parenting behavior” was chosen because it comes closest to the phenomenon of interest: concrete parenting practices that are shown repeatedly over the course of a person’s childhood and youth. In its meaning, it is close to the term “parenting style”, but it is not completely equivalent to it. While parenting styles include parents’ values, goals, and attitudes (Darling and Steinberg, 1993), parenting behavior as used in this study centers on concrete, observable actions.

When it comes to relationships between parents and their children, another concept that is important to explore is attachment. According to Benoit (2004), attachment can be defined as “one specific and circumscribed aspect of the relationship between a child and caregiver that is involved with making the child safe, secure and protected” (p. 541). John Bowlby was one of the most influential theorists in the field of attachment, with his work focusing on the development of attachment in infants (Bowlby, 1969, 1973, 1980). While early work on attachment focused on the relationships between children and their caregivers, a growing interest in studying attachment beyond early childhood has developed over time (Armsden and Greenberg, 1987). In a wider definition, attachment can be seen as “an enduring affectional bond of substantial intensity” (Armsden and Greenberg, 1987), not only appearing between children and their caretakers, but also between an individual and other people close to the individual, for example peers or partners (p. 429).

Now, is there a relationship between IWAH and parenting behavior, or between IWAH and attachment? The theoretical perspectives that inspired IWAH contain some ideas about possible relationships. Adler (2007/1927) believed that every person comes to the world with a certain degree of “Gemeinschaftsgefühl” (p. 54). Gemeinschaftsgefühl can be described as “a sense of belonging within and to the group” and as “a collective identity and shared endeavor” (La Voy et al., 2013, p. 281). Adler supposed that an individual’s Gemeinschaftsgefühl can expand over the life course (Adler, 2007/1927, p. 54). In his theory, a necessary precondition for this expansion is “a nurturing environment with a value for the other” (La Voy et al., 2013, p. 281). Adler also suspected that the expansion could be impeded by adverse circumstances, especially during childhood (Adler, 2007/1927, p. 39). He supposed that parenting behaviors preventing Gemeinschaftsgefühl from growing could be parents mollycoddling their children (“Verzärtelung”), treating them unlovingly (“Lieblosigkeiten”), and being too harsh (“harte Erziehung”; Adler, 2008/1933, p. 51).

Abraham Maslow believed so-called “self-actualized” individuals to “have for human beings in general a deep feeling of identification, sympathy, and affection” and “a genuine desire to help the human race,” “as if they were all members of a single family” (Maslow, 1954/1970, p. 165). However, he also believed that “very good conditions are needed to make self-actualizing possible,” also addressing familial conditions (Maslow, 1954/1970, p. 99). According to his theory, it is especially important for parents to satisfy their child’s safety needs (p. 39). He believed that “permissiveness within limits, rather than unrestricted permissiveness is preferred as well as needed by children” (Maslow, 1954/1970, p. 40). Also, he stated that a child’s need for safety could stay unfulfilled due to certain parental actions including “quarreling, physical assault, separation, divorce, or death within the family (…) parental outbursts of rage or threats of punishment directed to the child (…) or actual physical punishment” (Maslow, 1954/1970, p. 40). In his theory, the fulfillment of these needs is necessary to reach self-actualization (p. 99). Thus, according to his theory, unfavorable parenting behavior leading to unfulfilled safety needs can prevent self-actualization and the feelings of identification, sympathy, and affection toward the human family (p. 165).

McFarland et al. (2012) suggested that “early punitiveness and lack of affection appear to predispose one to be less concerned for all humanity, whereas a lack of punitiveness coupled with affection may provide a foundation for later concern for humanity at large” (p. 849). This concrete idea stems from the “Dual Process Model of Ideology and Prejudice” by Duckitt (2001). Part of this model is the idea that the development of RWA can be favored by a punitive socialization, while the development of SDO can be favored by an absence of childhood affection (Duckitt, 2001). As RWA and SDO show strong negative correlations with IWAH, a punitive socialization and the absence of affection in childhood might also negatively affect IWAH. Contradictory to this idea, an unpublished study by McFarland et al. (2013) found no relationship between IWAH and people’s memories of their parents’ childrearing. Besides, in another study, Hamer and McFarland (2018, June) found IWAH to be unrelated to harsh and strict socialization, as well as to unaffectionate socialization (McFarland et al., 2019).

However, an unpublished study by Hamer (2017) with an adult sample from Poland found that IWAH correlated significantly but weakly (0.08 to 0.21) with autonomy and acceptance given to children by their parents (McFarland et al., 2019). The same study also came to the result that IWAH correlated weakly positively with a secure attachment style and weakly negatively with a fearful one (McFarland et al., 2019). Moreover, Reysen and Katzarska-Miller (2013) found that a normative environment supporting global citizenship could predict global identification. In their study, such an environment consisted of people who were important to the surveyed person and found global citizenship desirable (p. 862).

### Aims of the present study

So far, only very few studies have analyzed the relationship between parenting behavior and IWAH and only one study has tried to examine the relationship between attachment and IWAH. Some of the most relevant findings in this field have not been published. The few published or cited studies have come to ambiguous results. Therefore, to this day, little is known about the relationship between the way a person has been raised and this person’s identification with and care for all humanity. Apart from that, theoretical ideas on the topic (Adler, 2007/1927, 2008/1933; Maslow, 1954/1970; Duckitt, 2001) are rather broad and stem from theories which all do not have parenting as their focus but rather mention it as a side issue. They primarily have in common that they suggest that positive parenting behavior might lead to IWAH while negative parenting behavior might prevent it from developing. Considering the lack of more concrete empirical and/or theoretical indications, the present study aimed to approach the relationship between parenting and IWAH by testing the following two rather unspecific hypotheses:

Hypothesis 1: There is a positive correlation between positive parenting behavior and IWAH, as well as between attachment and IWAH.

Hypothesis 2: There is a negative correlation between negative parenting behavior and IWAH.

An additional aim of the study was to explore the unique contribution of each facet of parenting when controlling for all other facets in a purely exploratory way.

## Materials and methods

### Design and procedure

The present study followed a correlational approach as described by Eid et al. (2017). The study’s survey was programmed with LimeSurvey (Limesurvey GmbH, 2012) and ran from June 25, 2021 to July 23, 2021. In this period, the respondi AG (2020), a company that provides online samples for social and market research and mainly recruits respondents via online campaigns (respondi AG, 2021), contacted participants from its German and English access panels. Choosing to sample from the access panels in Germany and England traces back to the study being part of a larger project with research questions concerning the European Union and Brexit. Before starting the survey, all participants gave informed consent following the criteria stated by the German Psychological Society [DGPs] (2016). Participants were informed that the survey contained questions about experienced negative parenting behavior and that it was recommended not to take part in the study if they did not want to expose themselves to these questions. While the survey was active, the five variables age (five groups), region (9 regions in England, 16 in Germany), gender, education (3 groups) and income (3 groups) were monitored by the respondi AG (2020). To create samples which are as representative as possible for the German and the English population, participants were contacted based on the quota of these five variables over the course of the study. Three items were integrated in the survey to check whether participants read the questions carefully or whether they just selected random answers (e.g., “Please select option 5 so that we can conduct data quality checks”). At the end of the survey, participants were informed about all constructs that were planned to be investigated using their data. For completing the survey, the participants received a small monetary reward.

### Participants

An a priori power analysis for the present study, which is described in detail in section 2.4.1, resulted in an optimal sample size of *n* = 1400. The respondi AG (2020) was commissioned to collect the data and to obtain the intended sample size. Finally, the data of 3056 participants were analyzed, 1517 from the German and 1539 from the English sample. Individuals had to be at least 18 years old to participate. The mean age of all participants who were included in the data analyses was 46.47 years (*SD* = 15.51). 50.34% were female, and almost half of the sample (45.41%) held at least an Access to Higher Education diploma (A level). Supplementary Table 1 (Supplementary Material 1) provides detailed information on the samples’ characteristics, separately as well as conjointly for the German and the English sample. Almost all participants answered the questions regarding remembered parenting behavior (RPB) and attachment for their biological parents. However, 7.95% of the participants also answered the questions for other people close to them, for instance their stepfathers or grandmothers. Supplementary Table 2 shows in detail for whom the participants assessed RPB and attachment.

### Measures

All participants were asked to fill out the following questionnaires. As the present study was embedded in a bigger study with other research topics, several other questionnaires were completed by the participants as well.

#### Identification with all humanity

Identification with all humanity was measured with the IWAH scale by McFarland et al. (2012) and its German translation by Reese et al. (2015). The scales can be found in Section 3 of the Supplementary Material. The original scale by McFarland et al. (2012) contains nine questions. Each of them is answered separately for three groups on a five-point scale (e.g., “1 = not at all” to “5 = very much”). As described by McFarland et al. (2012), the sum of all items forming the last group (e.g., “people all over the world”) constitutes the measure of IWAH. For the bigger study, two more groups (“Europeans” and “Followers of my religion/denomination”) were added to the IWAH scale. However, these groups were not analyzed in the present study.

The scale’s items have repeatedly been found to form two different factors with four items loading on each one (Reese et al., 2015; Reysen and Hackett, 2015; Sparkman and Hamer, 2020). Items 1–4 (e.g., “How often do you use the word “we” to refer to the following groups?”) usually load on a factor called “global self-definition” (GSD) or “bond,” while items 6–9 (e.g., “When they are in need, how much do you want to help people all over the world?”) usually load on a factor called “global self-investment” (GSI) or “concern” (Reese et al., 2015; Hamer et al., 2021). Item 5 usually cross-loads onto both factors as it incorporates parts of both GSD and GSI (Reese et al., 2015). Thus, previous studies came to the conclusion that this item might be dropped from analyses (Reese et al., 2015; Sparkman and Hamer, 2020).

Different studies found the IWAH scale to have good psychometric properties. The internal consistency of the total IWAH scale has been found to be acceptable to excellent in adult samples from different countries (α = 0.75 to α = 0.90; McFarland et al., 2012; Hamer et al., 2021). The internal consistency of the two facets GSD and GSI has been found to be acceptable to good (GSD: α = 0.73 to α = 0.86, GSI: α = 0.75 to α = 0.86; Reese et al., 2015; Hamer et al., 2021).

#### Remembered parenting behavior

The participants also answered several questions about the behavior their parents showed toward them over the course of their first 16 years of life. As these questions were retrospective, the overarching construct that was measured will from now on be called RPB. All questionnaires measuring RPB were presented twice, with one version measuring the participants’ mothers’ and one measuring their fathers’ RPB.

To measure negative RPB [henceforth negative remembered parenting behavior (nRPB)], twelve items from the “Measure of Parental Style” (MOPS) by Parker et al. (1997) and their German translation by Rumpold et al. (2002) were utilized. The MOPS consists of three facets, namely “Indifference” (e.g., “My mother/father was uninterested in me”), “Abuse” (e.g., “My mother/father made me feel in danger”), and “Over-Control” (e.g., “My mother/father was overprotective of me”). Four items of each facet were selected based on the factor loadings reported by Rumpold et al. (2002). As in the original MOPS, the perceived truth of all statements was rated on a four-point scale (from “1 = not true at all” to “4 = extremely true”). In the original English version of the MOPS, the internal consistencies of the three facets have been found acceptable to excellent (Indifference: α_mother_=α_father_=0.93; Abuse: α_mother_=0.87, α_father_=0.92; Over-Control: α_mother_=0.82, α_father_=0.76; Parker et al., 1997). In the German version, the internal consistencies of the facets Indifference and Abuse have been found acceptable to excellent (Indifference: α_mother_=0.87, α_father_=0.93, Abuse: α_mother_=0.84, α_father_=0.78; Rumpold et al., 2002). The internal consistency of the facet Over-Control has been found to be poor (α_mother_=0.58, α_father_=0.48; Rumpold et al., 2002). However, as only the four items with the highest factor loadings were selected, the facet was still included in the study.

Twelve other items were used to measure positive RPB [henceforth positive remembered parenting behavior (pRPB)]. These items were all either selected from one of the two following questionnaires or formulated by the authors of the study. The first questionnaire with items measuring pRPB was the “Evaluation des Pratiques Educatives Parentales” (EPEP; Evaluation of Educational Parental Practices) by Meunier and Roskam (2007). From the EPEP, the four items loading highest on the facet “Positive parenting” were selected (Meunier and Roskam, 2007). The EPEP being a questionnaire which is usually answered by parents, the chosen items were adapted to the child’s perspective and put into past tense (e.g., “My mother/father gave me a compliment, hug or a tap on the shoulder as a reward for good behavior”). As there was no German translation available, the questions were translated using the following back-translation method in combination with subsequent tests for measurement invariance, as recommended by van de Vijver and Poortinga (2005) and Schmitt and Eid (2007): One person translated the questions into German. Another person who had not seen the original questions before translated them back into English. At points where minor discrepancies between the original and the version that was translated back appeared, the translation was discussed until a consensus solution was found. Finally, an independent professional translator checked and approved, or adjusted the final version.

The second questionnaire with items measuring pRPB was the “Zürcher Kurzfragebogen zum Erziehungsverhalten” (ZKE; Zurich Short Questionnaire on Parental Behavior) by Reitzle et al. (2001). The four items loading highest on the facet “Warmth/Support” were selected, and again, adapted to the child’s perspective, and put into past tense (e.g., “My mother/father was there when I needed her/him”). Reports on the two questionnaires’ psychometric properties can be found in the studies by Meunier and Roskam (2007) and Reitzle et al. (2001). As only specific items instead of the original factors were used in the present study, previously reported internal consistencies of the original factors “Positive Parenting” and “Warmth/Support” are not restated here.

Additionally, four new items measuring parental love were formulated by the authors of the study (e.g., “My mother/father loved me”). Apart from the items being translated into English and then back into German, the translation process for these items was equal to the one already described for the EPEP items.

As all items measuring nRPB and pRPB were presented in one questionnaire, the scale measuring nRPB (“1 = not true at all” to “4 = extremely true”) was also used for the rating of all other items. Finally, the order of all items measuring RPB was modified to counteract possible position effects and response bias. Items measuring pRPB and nRPB were alternated. While the original order of the items measuring nRPB was maintained, items measuring the same facet of pRPB were placed as far away from each other as possible.

#### Attachment

Attachment was measured with a revised version of Armsden and Greenberg’s (1987) “Inventory of Parent and Peer Attachment” (IPPA). Items in the IPPA are usually rated on a five-point scale. However, as all questions concerning RPB were rated on a four-point scale in the present study, this four-point scale was also adopted for measuring attachment to avoid confusion. In its original version, the IPPA consists of three subscales, namely “Trust,” “Communication,” and “Alienation” (Armsden and Greenberg, 1987). In the present study, we only used the two positive subscales “Trust” and “Communication.” These two facets were thought of as valuable supplements to the facets concerning positive parenting behavior, as their items were close to parenting behavior as defined in our study (“concrete parenting practices that are shown repeatedly over the course of a person’s childhood and youth”), while still adding new aspects to it. While these two subscales had already been validated in a German sample and turned out to be reliable measures in a previous study (Bohn et al., 2020), the negative subscale “Alienation” had neither been translated, nor validated so far. Thus, we decided to drop this subscale. Hence, four items were selected from each of the two subscales “Communication” (e.g., “My mother/father helped me to talk about my difficulties”) and “Trust” (e.g., “My mother/father trusted my judgement”). The item selection was based on the items’ factor loadings in Armsden and Greenberg’s (1987) original version of the IPPA, as item loadings for the revised version were not available. The German items stem from a translation by Renate Baudis (Greenberg, 2021, personal communication). They were used and rewritten from the third person plural into the third person singular with kind permission of Mark T. Greenberg. Again, as only specific items were used in the present study, previously reported internal consistencies of the original factors “Trust” and “Communication” are not restated here.

### Statistical analysis

Statistical analyses were conducted in December 2021 with R (version 4.0.4) and RStudio (version 1.4.1106; R Core Team, 2021). Following prevalent conventions, the significance level for all analyses was set to α=0.05, unless stated otherwise.

#### A priori power analysis

An a priori power analysis was conducted with the Shiny App “pwrSEM” (Wang and Rhemtulla, 2021) to determine the sample size that is necessary to assure an adequate power. The app works with a Monte Carlo simulation approach and allows to calculate power to detect a target effect in structural equation modeling (SEM; Wang and Rhemtulla, 2021). For this study, the power analysis aimed to assure a power of 0.80 for the correct rejection of all possible null hypotheses that certain correlations in the population are zero, given specific values for these correlations.

**FIGURE 1:**
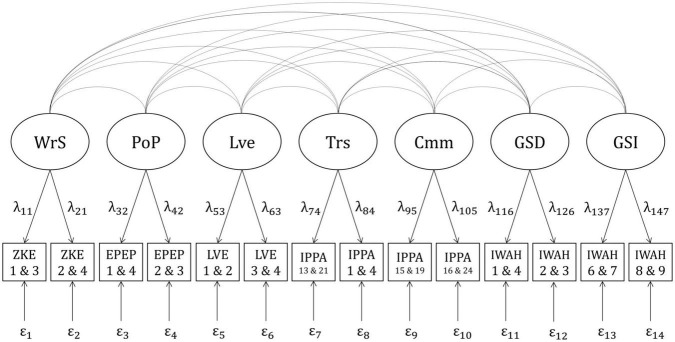
Positive model with correlations between positive RPB, attachment, and IWAH. WrS, Warmth/Support; PoP, positive parenting; Lve, love; Trs, trust; Cmm, communication; GSD, global self-definition; GSI, global self-investment; ZKE, “Zürcher Kurzfragebogen zum Erziehungsverhalten”; EPEP, “Evaluation des Pratiques Educatives Parentales”; LVE, items measuring parental love; IPPA, “Inventory of Parent and Peer Attachment”; IWAH, “Identification With All Humanity scale”. Latent factors (e.g., WrS) are displayed in circles. Indicators (e.g., ZKE 1 and 3) are displayed in squares. Numbers beneath the abbreviations of questionnaires indicate item numbers of the items forming particular indicators (e.g., Item 1 and item 3 from the ZKE form one indicator displayed as ZKE 1 and 3). Curved lines indicate correlations between latent factors. Arrows with factor loadings (e.g., λ_11_) illustrate the assumption that the indicators are predicted by specific latent factors. Residual variances are displayed below the indicators (e.g., ε_1_).

**FIGURE 2:**
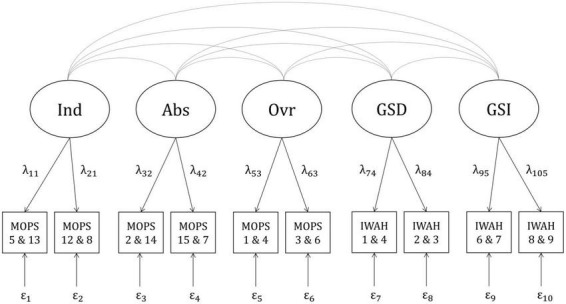
Negative model with correlations between negative RPB and IWAH. Ind, indifference; Abs, abuse; Ovr, over-control; GSD, global self-definition; GSI, global self-investment; MOPS, “Measure of Parental Style”; IWAH, “Identification With All Humanity scale”. Latent factors (e.g., Ind) are displayed in circles. Indicators (e.g., MOPS 5 and 13) are displayed in squares. Numbers beneath the abbreviations of questionnaires indicate item numbers of the items forming particular indicators (e.g., Item 5 and item 13 from the MOPS form one indicator displayed as MOPS 5 and 13). Curved lines indicate correlations between latent factors. Arrows with factor loadings (e.g., λ_11_) illustrate the assumption that the indicators are predicted by specific latent factors. Residual variances are displayed below the indicators (e.g., ε_1_).

The smallest value for a correlation to be regarded as relevant was *r* = |0.20|. Therefore, the correlations between each facet of pRPB and both facets of IWAH, as well as between both facets of attachment and both facets of IWAH were set to 0.20. Correlations between each facet of nRPB and both facets of IWAH were set to −0.20. As correlations between facets of nRPB and pRPB, as well as between facets of nRPB and attachment were difficult to estimate prior to the study, the power analysis was split into two parts. The first part included correlations between all facets of pRPB, attachment, and IWAH (positive model, see Figure 1). The second part included correlations between all facets of nRPB and IWAH (negative model, see Figure 2). Each facet in both models was planned to be measured by four items. The four items corresponding to one facet were split into two parcels consisting of two items each. Correlations between the different facets of RPB and attachment, as well as the loadings of the observed variables were estimated based on the questionnaires’ manuals and studies investigating similar variables (Armsden and Greenberg, 1987; Parker et al., 1997; Reitzle et al., 2001; Schönpflug, 2001; Rumpold et al., 2002; Meunier and Roskam, 2007; Reese et al., 2015; Pastorelli et al., 2016; Wong et al., 2020). Residual variances of the observed variables were calculated as follows (Eid et al., 2017):


(1) $$V\,a\,r\,\left(\varepsilon_{\text{i}}\right)=V\,a\,r\,\left(Y_{\text{i}}\right)-\lambda_{\text{i}}^{2}\cdot V\,a\,r\,\left(\eta \right)$$

The seed for simulations as well as the number of simulations were set to 10,000. The final values entered in the power analyses can be found in Supplementary Table 4 (positive model) and Supplementary Table 5 (negative model). Systematic variation of possible sample sizes led to the result that assuring a power of 0.80 for the correct rejection of all null hypotheses of correlations being zero in the population required a sample size of *n* ≈ 700 in the negative and of *n* ≈ 580 in the positive model. As an adequate power needed to be assured for both models, as well as for the German and the English sample, the power analysis resulted in a necessary sample size of at least *n* = 700 per sample.

Other research questions were planned to be investigated with the same dataset. Power analyses for the corresponding models resulted in an optimal sample size of *n* ≈ 1350 per sample. As the data collection was based on this size, an adequate power for the present study was clearly ensured.

#### Data preparation

The R packages “tidyverse” (Wickham et al., 2019), “psych” (Revelle, 2020), and “naniar” (Tierney et al., 2021) were used to prepare the data for further analyses. Already factoring in dropout, the respondi AG (2020) exceeded the necessary sample size of *n* = 1350 per sample in the data collection process. Hence, the raw dataset consisted of 6591 participants (2998 German and 3593 English). For all our further analyses, we then excluded participants that had (a) not accepted the conditions of participation, (b) dropped out before having been presented with all questionnaires relevant to the study, or (c) given no answers at all for the items measuring RPB and attachment. To ensure basing our analyses on carefully and honestly completed questionnaires only, we also excluded participants that (d) had failed to answer the first quality check item correctly^1^ or (e) for whom we had found more than twice as many further hints of poor data quality than on average (relative to the number of questionnaire pages completed). Such hints included speeding, i.e., answering long pages very quickly, straightlining, i.e., ticking the same answer option across an entire long page, or clearly answering semantically unrelatedly to a free-text item concerning a spiritual life event (see Bowling, 2005; Zhang and Conrad, 2014; Callegaro et al., 2015). We diversified our criteria to alleviate weaknesses some of them show in the detection of suspicious observations (Reuning and Plutzer, 2020). Note that only the presence of at least four hints led to exclusion from the sample, while, e.g., ticking the same answer option across an entire page only on one or two pages did not (see above). Altogether, we excluded 1481 German and 2054 English participants based on these five criteria, resulting in final sample sizes of *n* = 1517 for the German, and *n* = 1539 for the English sample.

#### Confirmatory factor analyses

To incorporate the effects of measurement error into the statistical analyses, SEM were applied. The relevant models, which were derived from theoretical assumptions and previous studies, were already introduced in the Chapter “a priori power analysis”. They are displayed in Figure 1 (positive model) and Figure 2 (negative model). One confirmatory factor analysis (CFA) was conducted for each combination of sample (German vs. English) × model (positive vs. negative), resulting in four separate analyses. The CFA were analyzed with the R package “lavaan” (Rosseel, 2012) using full information robust maximum likelihood estimation (MLR), which does not require multivariate normal distribution of the observed variables. The evaluation of the four CFA was based on the models’ chi-square statistics (χ^2^), Standardized Root Mean Square Residuals (*SRMR*), Comparative Fit Indices (*CFI*), and Root Mean Square Error(s) of Approximation (*RMSEA*). The criteria for good model fit followed the recommendations of Schermelleh-Engel et al. (2003): A ratio of χ^2^/*df* ≤ 2, ideally with a non-significant χ^2^(*p* > 0.05), *SRMR* ≤ 0.05, *CFI*≥0.97, and *RMSEA* ≤ 0.05. Composite reliabilities of the factors (McDonald’s ω) were calculated with the reliability() function in the R package “semTools” (Jorgensen et al., 2022). For factors with cross-loadings, composite reliabilities were calculated using formula (2) in Raykov and Shrout (2002).

#### Measurement invariance

To test the psychometric equivalence of the latent variables, measurement invariance across the two samples (German and English) was tested for both models (positive and negative). The measurement invariance was tested by calculating Satorra-Bentler scaled χ^2^ difference tests (Satorra and Bentler, 2001) in each of which the fit of two nested models (e.g., one configural and one metric invariance model) was compared. Increasingly restrictive models were tested until a significant difference between two models was observed. All tests concerning measurement invariance were conducted with the R package “lavaan” (Rosseel, 2012).

#### Correlation analyses

As different facets of positive and negative parenting behavior were measured, the study’s hypotheses concerning correlations were specified as follows:

Hypothesis 1: There is a positive correlation between each facet of positive parenting behavior and both facets of IWAH, as well as between each facet of attachment and both facets of IWAH.

Hypothesis 2: There is a negative correlation between each facet of negative parenting behavior and both facets of IWAH.

Structural equation models were used to test these two hypotheses, as they allow to estimate correlations between latent variables which are free from measurement error. To separate measurement error from true individual differences we used the parceling approach (see, e.g., Kline, 2011) with two indicators (split-half procedure) for each factor. The models depicted in Figures 1, 2 were used to test the two hypotheses separately for the German and the English sample. Thus, four model comparisons were conducted. In each comparison, two kinds of models were compared: A first model which allowed for correlations between all latent variables included in the model, and a second, more restricted model, in which all correlations of interest were set to zero. Satorra-Bentler scaled χ^2^ difference tests (Satorra and Bentler, 2001), which are robust to violations of multivariate normality, and Bayesian Information Criteria (*BIC*) were calculated to compare the fit of the two models.

Based on the result of each χ^2^ difference test and on the *BIC*, a decision for one of the models was made. A significant difference according to the χ^2^ difference test in conjunction with the model allowing for all correlations having a lower *BIC* led to a decision for the respective model. If all estimated correlations had the expected sign (positive signs for Hypothesis 1, negative for Hypothesis 2), the corresponding hypothesis was accepted. Finally, the significance of single correlations in the analyzed model was tested. To account for the alpha error accumulation occurring in multiple testing, the False Discovery Rate (*FDR*) was controlled by calculating adjusted *p*-values after Benjamini and Hochberg (1995). Whenever the models did not have a significantly differing fit according to the χ^2^ difference test and whenever the model in which all correlations were set to zero had a lower *BIC*, the decision was made in favor of the more restricted model. In these cases, the respective hypothesis was rejected.

#### Regression analyses

First, to estimate the multiple correlation between the facets of parenting and IWAH (square root of coefficient of determination) and to test whether this multiple correlation is significantly different from 0, and second, to explore the unique contribution of each facet of parenting when controlling for all other facets in a purely exploratory way, we calculated latent regression analyses.

Hence, the factors representing IWAH (dependent variables, GSD and GSI) were regressed on the factors representing RPB and attachment. Regression analyses were only conducted when a model allowing for all correlations was preferred over a model in which all correlations were set to zero in the corresponding correlation analysis. For each regression model, the significance of single regression coefficients was tested and the percentage of variance in the dependent variables (GSD and GSI) which could be explained by the relevant latent variables (*R*^2^) as well as the multiple correlation between the facets of parenting and IWAH (*R*) were calculated.

Beside analyzing the positive and the negative models separately, one regression analysis was calculated per sample, in which all positive and negative facets were included as predictors. For these regressions, too, the percentage of variance in the dependent variables (GSD and GSI) which could be explained by all positive and negative facets (*R*^2^) as well as the multiple correlation between all positive and negative facets and IWAH (*R*) were calculated.

## Results

### Descriptive statistics

Density plots of the study’s most important variables are displayed in Supplementary Figure 1 (GSD and GSI), Supplementary Figure 2 (pRPB variables: Warmth/Support, Positive Parenting, and Love), Supplementary Figure 3 (nRPB variables: Indifference, Abuse, and Over-Control), and Supplementary Figure 4 (attachment variables: Communication and Trust). Examining the plots leads to two key findings. Firstly, the variables’ distributions only show small differences between the German and the English sample. In the English sample, the means of the nRPB facets Indifference and Abuse are lower, the mean of GSD is slightly lower, and the means of all pRPB and attachment facets are slightly higher than in the German sample. And secondly, all nRPB variables are clearly positively skewed in both samples, which indicates that most participants reported not having experienced much negative parenting behavior. Means, standard deviations, and manifest correlations between the study’s main variables can be found in Table 1 (German sample) and Table 2 (English sample).

**TABLE 1:** Means, standard deviations, and bivariate correlations for the German sample.

	*M (SD)*	GSD	GSI	WrS	PoP	Love	Cmm	Trs	Ind	Abs
GSD	9.75 (3.42)	1								
GSI	13.04 (4.05)	0.68***	1							
WrS	2.82 (0.85)	0.11***	0.14***	1						
PoP	2.44 (0.86)	0.15***	0.15***	0.90***	1					
Love	2.92 (0.86)	0.10***	0.12***	0.94***	0.85***	1				
Cmm	2.36 (0.93)	0.16***	0.11***	0.83***	0.87***	0.81***	1			
Trs	2.69 (0.87)	0.12***	0.11***	0.88***	0.85***	0.89***	0.88***	1		
Ind	1.69 (0.72)	−0.02	−0.05	−0.69***	−0.59***	−0.72***	−0.57***	−0.68***	1	
Abs	1.63 (0.66)	0.04	0.01	−0.60***	−0.50***	−0.64***	−0.50***	−0.63***	0.81***	1
Ovr	1.78 (0.57)	0.08**	0.03	−0.32***	−0.25***	−0.36***	−0.28***	−0.42***	0.55***	0.71***

N = 1478 to 1516. GSD, global self-definition (scale: 4 to 20); GSI, global self-investment (scale: 4 to 20); WrS, Warmth/Support; PoP, positive parenting; Cmm, communication; Trs, trust; Ind, indifference; Abs, abuse; Ovr, over-control. Scales for all variables apart from GSI and GSD range from 1 to 4. **p < 0.01. ***p < 0.001.

**TABLE 2:** Means, standard deviations, and bivariate correlations for the English sample.

	*M (SD)*	GSD	GSI	WrS	PoP	Love	Cmm	Trs	Ind	Abs
GSD	9.38 (3.43)	1								
GSI	13.43 (4.06)	0.59***	1							
WrS	2.85 (0.85)	0.11***	0.15***	1						
PoP	2.50 (0.86)	0.16***	0.17***	0.90***	1					
Love	3.00 (0.86)	0.09***	0.13***	0.93***	0.85***	1				
Cmm	2.42 (0.93)	0.16***	0.11***	0.82***	0.84***	0.78***	1			
Trs	2.76 (0.87)	0.12***	0.11***	0.86***	0.90***	0.85***	0.87***	1		
Ind	1.50 (0.72)	0.07**	−0.02	−0.61***	−0.49***	−0.66***	−0.46***	−0.57***	1	
Abs	1.37 (0.66)	0.13***	0.06*	−0.46***	−0.35***	−0.52***	−0.32***	−0.45***	0.79***	1
Ovr	1.75 (0.57)	0.15***	0.11***	−0.29***	−0.22***	−0.34***	−0.25***	−0.41***	0.54***	0.65***

N = 1512 to 1539. GSD, global self-definition (scale: 4 to 20); GSI, global self-investment (scale: 4 to 20); WrS, Warmth/Support; PoP, positive parenting; Cmm, communication; Trs, trust; Ind, indifference; Abs, abuse; Ovr, over-control. Scales for all variables apart from GSI and GSD range from 1 to 4. *p < 0.05. **p < 0.01. ***p < 0.001.

### Confirmatory factor analyses

#### Positive model, German sample

**FIGURE 3:**
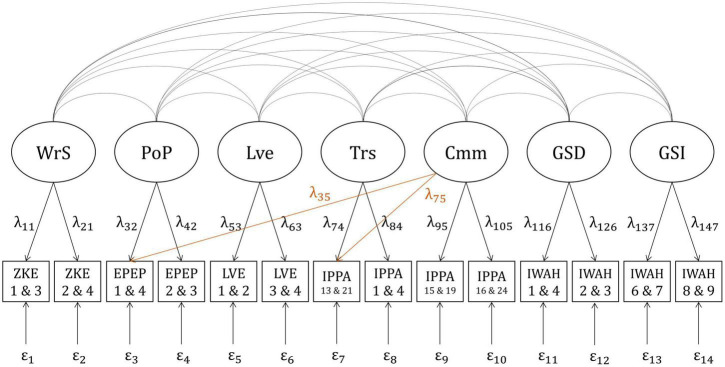
Adjusted positive correlation model for the German and the English sample. WrS, Warmth/Support; PoP, positive parenting; Lve, love; Trs, trust; Cmm, communication; GSD, global self-definition; GSI, global self-investment; ZKE, “Zürcher Kurzfragebogen zum Erziehungsverhalten”; EPEP, “Evaluation des Pratiques Educatives Parentales”; LVE, items measuring parental love; IPPA, “Inventory of Parent and Peer Attachment”; IWAH, “Identification With All Humanity scale”. Latent factors (e.g., WrS) are displayed in circles. Indicators (e.g., ZKE 1 and 3) are displayed in squares. Numbers beneath the abbreviations of questionnaires indicate item numbers of the items forming particular indicators (e.g., Item 1 and item 3 from the ZKE form one indicator displayed as ZKE 1 and 3). Curved lines indicate correlations between latent factors. Arrows with factor loadings (e.g., λ_11_) illustrate the assumption that the indicators are predicted by specific latent factors. Residual variances are displayed below the indicators (e.g., ε_1_). One modification of the original positive model (Figure 1) was made to improve model fit: the factor “communication” was allowed to additionally load on the indicators “IPPA 13 and 21” and “EPEP 1 and 4.” This change is marked in orange.

The positive model (Figure 1) did not have an acceptable fit in the German sample: χ^2^(56) = 465.966, *p* < 0.001; *SRMR* = 0.014; *CFI* = 0.981; *RMSEA* = 0.075, 90%*CI*[0.069,0.081]. Therefore, the model’s modification indices were examined. The indices suggested that the model’s fit could be improved by allowing the two indicators “IPPA 13 and 21” and “EPEP 1 and 4” to have additional loadings on the factor “Communication”. The model was adjusted accordingly. Modifying the model led to a new model which fit the data well: χ^2^(54) = 235.783, *p* < 0.001; *SRMR* = 0.009; *CFI* = 0.992; *RMSEA* = 0.050,90%*CI*[0.044,0.057]. Reliabilities of the indicators ranged from 0.744 (IWAH 1 and 4) to 0.956 (IPPA 1 and 4) and composite reliabilities of the factors ranged from 0.869 (GSD) to 0.965 (Trs; see Supplementary Table 6). The adjusted model displayed in Figure 3 was therefore used in all further analyses.

#### Positive model, English sample

In the English sample, the original positive model had a poor fit as well: χ^2^(56) = 494.089, *p* < 0.001; *SRMR* = 0.017; *CFI* = 0.980; *RMSEA* = 0.075,90%*CI*[0.069,0.081]. Hence, the model was modified the same way as in the German sample. The adjusted model (Figure 3) had an acceptable fit: χ^2^(54) = 284.340, *p* < 0.001; *SRMR* = 0.013; *CFI* = 0.990; *RMSEA* = 0.055,90%*CI*[0.049,0.062]. Reliabilities of the indicators ranged from 0.636 (IWAH 1 and 4) to 0.937 (IPPA 1 and 4) and composite reliabilities of the factors ranged from 0.819 (GSD) to 0.956 (Trs; see Supplementary Table 7). Thus, this model was used in all further analyses.

#### Negative model, German sample

The negative model (Figure 2) fit the data from the German sample well by all criteria: χ^2^(25) = 64.026, *p* < 0.001; *SRMR* = 0.025; *CFI* = 0.996; *RMSEA* = 0.033, 90%*CI*[0.023,0.043]. Reliabilities of almost all indicators ranged from 0.738 (IWAH 1 and 4) to 0.832 (MOPS 12 and 8). One indicator had a very low reliability of 0.356 (MOPS 1 and 4). As composite reliabilities of all factors ranged from 0.726 (Ovr) to 0.891 (Abs; see Supplementary Table 8), the modified model was used in all further analyses.

#### Negative model, English sample

The negative model had a good fit in the English sample, too: χ^2^(25) = 110.226, *p* < 0.001; *SRMR* = 0.029; *CFI* = 0.991; *RMSEA* = 0.050,90%*CI*[0.041,0.060]. Reliabilities of the indicators ranged from 0.624 (IWAH 1 and 4) to 0.930 (MOPS 12 and 8). Just as in the German sample, the indicator MOPS 1 and 4 had a very low reliability of 0.457. However, the composite reliabilities of all factors ranged from 0.816 (Ovr) to 0.952 (Ind; see Supplementary Table 9), so the modified model was used in all further analyses of the English sample as well.

### Measurement invariance

#### Positive model

Measurement invariance across the two samples was tested based on the previously adjusted model (Figure 3). A first χ^2^-difference test was conducted to compare the model fits of the following two models: (a) a configural model (χ^2^(108) = 519.296, *p* < 0.001; *SRMR* = 0.011; *CFI* = 0.991; *RMSEA* = 0.053,90%*CI*[0.048,0.058]) which only assumed that the latent variables in the positive model (GSD, GSI, WrS, PoP, Lve, Trs, and Cmm) had the same pattern of free and fixed loadings in the German as in the English sample and (b) a metric model (χ^2^(117) = 532.188, *p* < 0.001; *SRMR* = 0.012; *CFI* = 0.991; *RMSEA* = 0.051,90%*CI*[0.046,0.055]) which also assumed that the factor loadings were equivalent in the two groups. This first test was not significant △χ^2^(9) = 7.6512, *p* = 0.57. The non-significant result allows us to infer that metric invariance between the two samples was likely given (Putnick and Bornstein, 2016).

A second χ^2^-difference test was conducted to compare the model fits of the previously tested metric model (χ^2^(117) = 532.188, *p* < 0.001; *SRMR* = 0.012; *CFI* = 0.991; *RMSEA* = 0.051,90%*CI*[0.046,0.055]) and a scalar model (χ^2^(124) = 759.082, *p* < 0.001; *SRMR* = 0.016; *CFI* = 0.986; *RMSEA* = 0.061,90%*CI*[0.057,0.065]) which additionally assumed that the item intercepts were equivalent in the German and the English sample. This test turned out to be significant △χ^2^(7) = 246.06, *p* < 0.001. Therefore, scalar invariance was not given for the positive model. All further analyses were conducted separately for the German and the English sample.

#### Negative model

Just as for the positive model, a first χ^2^-difference test was conducted to compare the model fits of a configural model (χ^2^(50) = 175.859, *p* < 0.001; *SRMR* = 0.027; *CFI* = 0.993; *RMSEA* = 0.043,90%*CI*[0.036,0.050]) and a metric model (χ^2^(55) = 208.665, *p* < 0.001; *SRMR* = 0.030; *CFI* = 0.992; *RMSEA* = 0.045,90%*CI*[0.039,0.052]). This test turned out to be significant △χ^2^(5) = 31.521, *p* < 0.001. Thus, metric invariance was not given for the negative model. However, as configural invariance was ensured through the preceding CFA and as the study did not contain group difference tests, the metric non-invariance was not a problem for the further analyses.

### Latent correlation analyses

Correlations between all latent variables for both samples are displayed in Table 3 (positive model) and Table 4 (negative model). To begin with, the tables show strong positive correlations between all facets of pRPB and attachment (0.84 to 1), between the facets of nRPB (0.65 to 0.98), and between GSD and GSI (0.69 to 0.78). These correlations are all in line with prior expectations.

**TABLE 3:** Latent correlations in the positive model.

	GSD	GSI	WrS	PoP	Love	Cmm	Trs
GSD	1	0.774***	0.128***	0.173***	0.115***	0.180***	0.117***
GSI	0.692***	1	0.155***	0.176***	0.137***	0.128***	0.097**
WrS	0.127***	0.169***	1	0.970***	0.996***	0.876***	0.912***
PoP	0.180***	0.193***	0.971***	1	0.910***	0.907***	0.845***
Love	0.108***	0.154***	1.011***	0.933***	1	0.847***	0.921***
Cmm	0.183***	0.120***	0.881***	0.881***	0.843***	1	0.859***
Trs	0.119***	0.115***	0.904***	0.838***	0.911***	0.851***	1

Correlations above the diagonal stem from the German sample, those below the diagonal stem from the English sample. N = 1517 (German sample) and 1539 (English sample). GSD, global self-definition; GSI, global self-investment; WrS, Warmth/Support; PoP, positive parenting; Cmm, communication; Trs, trust. ***p < 0.001.

**TABLE 4:** Latent correlations in the negative model.

	GSD	GSI	Ind	Abs	Ovr
GSD	1	0.775***	−0.017	0.030	0.042
GSI	0.690***	1	−0.046	0.017	0.007
Ind	0.058	−0.040	1	0.981***	0.712***
Abs	0.153***	0.053	0.844***	1	0.905***
Ovr	0.151***	0.088*	0.648***	0.761***	1

Correlations above the diagonal stem from the German sample, those below the diagonal stem from the English sample. N = 1517 (German sample) and 1539 (English sample). GSD, global self-definition; GSI, global self-investment; Ind, indifference; Abs, abuse; Ovr, over-control. *p < 0.05. ***p < 0.001.

In both samples, all facets of attachment and pRPB correlate weakly positively (0.10 to 0.19) with the two facets of IWAH. These correlations can be interpreted as a first indicator of the possible acceptance of Hypothesis 1. Contrarily, the correlations concerning Hypothesis 2, which are the correlations between the facets of nRPB and IWAH, are predominantly close to zero and non-significant, which distinguishes them from all other correlations. In the English sample, three correlations between facets of nRPB and IWAH are significant. Interestingly, they are all weakly positive (*r*_*GSD*, *Abs*_=0.15, *r*_*GSD*, *Ovr*_=0.15, *r*_*GSI*, *Ovr*_=0.09). Thus, Hypothesis 2 is likely to be rejected.

Table 5 contains chi-square values (χ^2^) and *BIC* for all models which were tested in the study’s correlation analyses.

**TABLE 5:** Test statistics of all eight models and results of the four χ^2^-difference tests.

Test statistics	Hypothesis 1, positive models				Hypothesis 2, negative models			
	German sample		English sample		German sample		English sample	
	Cor. allowed	Cor. zero	Cor. allowed	Cor. zero	Cor. allowed	Cor. zero	Cor. allowed	Cor. zero
*N*	1517	1517	1539	1539	1517	1517	1539	1539
χ^2^	235.783	314.497	284.340	380.434	64.026	77.137	110.226	165.051
*df*	54	64	54	64	25	31	25	31
*p*	<0.001***	<0.001***	<0.001***	<0.001***	<0.001***	<0.001***	<0.001***	<0.001***
*BIC*	36248	36260	38058	38089	33527	33497	32428	32443
χ^2^-diff.	81.985		97.191		13.11		57.248	
*df*-diff.	10		10		6		6	
*p*	<0.001***		<0.001***		0.04*		<0.001***	

“Cor. allowed” describes the models allowing for correlations between all latent variables. “Cor. zero” describes the models in which all correlations of interest were set to zero. The row “χ^2^” contains robust chi-square values for all models, the row “BIC” contains the models’ robust Bayesian Information Criteria. “χ^2^-diff.” is the test statistic for the scaled χ^2^-difference test. “*df*-diff.” is the corresponding *df*-difference. Note that as the χ^2^-difference test is a function of two standard (not robust) test statistics, the χ^2^-difference test statistic cannot be calculated by simply subtracting the scaled χ^2^ test statistics above. *p < 0.05. ***p < 0.001.

#### Testing Hypothesis 1

The following models were compared to test Hypothesis 1: a first model which allowed for correlations between all facets of pRPB and IWAH as well as between all facets of attachment and IWAH, and a second model which was a more restricted version of the first model in which correlations between all facets of pRPB and IWAH as well as between all facets of attachment and IWAH were set to zero. For the German as well as for the English sample, the previously adjusted model displayed in Figure 3 was used as a basis for the two different models.

##### Results in the German sample

The first χ^2^ difference test revealed that the two positive models differed significantly regarding their model fit: χ^2^(10) = 81.985, *p* < 0.001. Moreover, the model allowing for all correlations had a lower *BIC* than the other (see Table 5). This indicates that the model allowing for all correlations describes the relationships between the latent variables better when considering model fit as well as parsimony. Therefore, the model allowing for all correlations was preferred over the model in which all correlations of interest were set to zero. The estimated correlations between the two facets of IWAH and all facets of pRPB and attachment were all positive and ranged from *r*_*GSI*, *Trs*_=0.097to *r*_*GSD*, *Cmm*_=0.180 (see Table 3). Without correcting for alpha error accumulation, all correlations of interest were significant on a level of α=0.001. When controlling the *FDR* by calculating adjusted *p*-values according to Benjamini and Hochberg (1995), all correlations remained significant. Thus, the hypothesis that there is a positive correlation between each facet of positive parenting behavior and both facets of IWAH, as well as between each facet of attachment and both facets of IWAH was accepted for the German sample. All estimated parameters of the positive model allowing for all correlations in the German sample can be found in Supplementary Table 6.

##### Results in the English sample

The second χ^2^ difference test revealed that the model allowing for all correlations fit the data significantly better: χ^2^(10) = 97.191, *p* < 0.001 (see Table 5). As the model allowing for all correlations also had a lower *BIC*, it was preferred over the other. Just as in the German sample, the correlations between the two facets of IWAH and all facets of pRPB and attachment were positive. In the English sample, they ranged from *r*_*GSD*, *Lve*_=0.108to *r*_*GSI*, *PoP*_=0.193. All these correlations were significant on a level of α=0.05. When calculating adjusted *p*-values according to Benjamini and Hochberg (1995), all correlations remained significant. Thus, the hypothesis that there is a positive correlation between each facet of positive parenting behavior and both facets of IWAH, as well as between each facet of attachment and both facets of IWAH was also accepted for the English sample. The estimated parameters of the positive model allowing for all correlations in the English sample can be found in Supplementary Table 7.

#### Testing Hypothesis 2

Two other types of models were compared to test Hypothesis 2: A first model which allowed for correlations between all facets of nRPB and IWAH, and a second model which was a more restricted version of the first model in which correlations between all facets of nRPB and IWAH were set to zero. The negative model in Figure 2 was used as a basis for the two different models in both samples.

##### Results in the German sample

The third χ^2^ difference test revealed that the two negative models differed significantly regarding their model fit: χ^2^(6) = 13.11, *p* < 0.04 (see Table 5). However, as the model in which all correlations were set to zero had a lower *BIC*, this model was preferred over the model allowing for all correlations. A closer look at the estimated correlations between the facets of nRPB and the facets of IWAH also revealed that all of them were very small (ranging from *r*_*GSI*, *Ind*_ = -0.046 to *r*_*GSD*, *Ovr*_=0.042) and none of them were significant. Therefore, the hypothesis that there is a negative correlation between each facet of negative parenting behavior and both facets of IWAH was rejected for the German sample. All estimated parameters of the negative model allowing for all correlations in the German sample can be found in Supplementary Table 8.

##### Results in the English sample

The fourth χ^2^ difference test revealed that the model allowing for correlations between all facets of IWAH and nRPB fit the data significantly better than the alternative model: χ^2^(6) = 57.248, *p* < 0.001 (see Table 5). The model which allowed for all correlations also had a lower *BIC*. Therefore, the model allowing for all correlations was preferred over the model in which all correlations of interest were set to zero. Out of the six correlations between all facets of IWAH and nRPB, three correlations were significant on a level of α=0.05 when not correcting for alpha error accumulation (*r*_GSD,Abs_, *r*_GSD,Ovr_ and *r*_GSI,Ovr_). When calculating adjusted *p*-values according to Benjamini and Hochberg (1995), these three correlations remained significant. However, as half of the correlations were not significant, the hypothesis that there is a negative correlation between *each* facet of negative parenting behavior and both facets of IWAH also needed to be rejected for the English sample. In addition to that, surprisingly, all significant correlations were positive *(r*_*GSD*, *Abs*_=0.153, *r*_*GSD*, *Ovr*_=0.151 and *r*_*GSI*, *Ovr*_=0.088). To further analyze the unexpected data structure in the negative model for the English sample, it was included in the subsequent regression analyses as well. All estimated parameters of the negative model allowing for all correlations in the English sample can be found in Supplementary Table 9.

### Regression analyses

**FIGURE 4:**
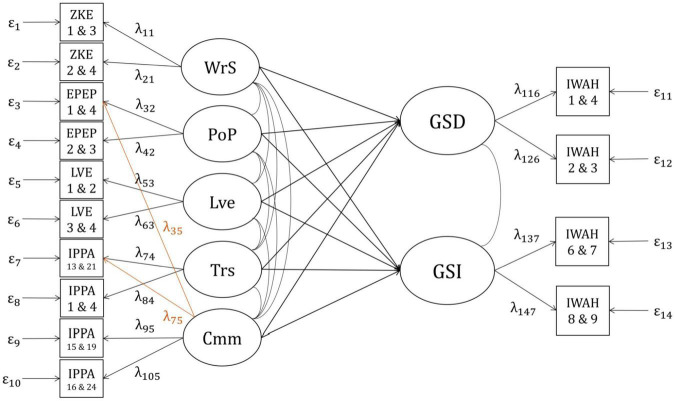
Positive regression model for the German and the English sample. WrS, Warmth/Support; PoP, positive parenting; Trs, trust; Cmm, communication; GSD, global self-definition; GSI, global self-investment; ZKE, “Zürcher Kurzfragebogen zum Erziehungsverhalten”; EPEP, “Evaluation des Pratiques Educatives Parentales”; LVE, items measuring parental love; IPPA, “Inventory of Parent and Peer Attachment”; IWAH, “Identification With All Humanity scale.” Latent factors (e.g., WrS) are displayed in circles. Indicators (e.g., ZKE 1 and 3) are displayed in squares. Numbers beneath the abbreviations of questionnaires indicate item numbers of the items forming particular indicators (e.g., Item 1 and item 3 from the ZKE form one indicator displayed as ZKE 1 and 3). Arrows with factor loadings (e.g., λ_11_) illustrate the assumption that the indicators are predicted by specific latent factors. Residual variances are displayed left and right to the indicators (e.g., ε_1_). Curved lines indicate correlations between latent factors. Arrows from latent variables to other latent variables (e.g., WrS → GSD) illustrate the idea that one variable on which the arrow points (e.g., GSD) is predicted by another (e.g., WrS). The change from the original model in Figure 1, which was modified slightly in the CFA, is marked in orange. In contrast to the correlation model which resulted from the CFA, in the present predictive model, the two facets of IWAH (GSD and GSI) are regressed on the variables representing pRPB and attachment.

**FIGURE 5:**
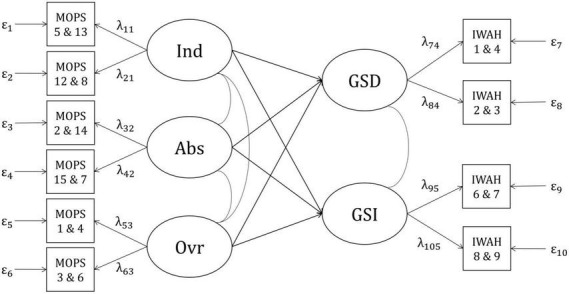
Negative regression model for the English sample. Ind, indifference; Abs, abuse; Ovr, over-control; GSD, global self-definition; GSI, global self-investment; MOPS, “Measure of Parental Style”; IWAH, “Identification With All Humanity scale.” Latent factors (e.g., Ind) are displayed in circles. Indicators (e.g., MOPS 5 and 13) are displayed in squares. Numbers beneath the abbreviations of questionnaires indicate item numbers of the items forming particular indicators (e.g., Item 5 and item 13 from the MOPS form one indicator displayed as MOPS 5 and 13). Curved lines indicate correlations between latent factors. Arrows with factor loadings (e.g., λ_11_) illustrate the assumption that the indicators are predicted by specific latent factors. Residual variances are displayed below the indicators (e.g., ε_1_). Arrows from latent variables to other latent variables (e.g., Ind → GSD) illustrate the idea that one variable on which the arrow points (e.g., GSD) is predicted by another (e.g., Ind). In contrast to the correlation model, in the present predictive model, the two facets of IWAH (GSD and GSI) are regressed on the variables representing nRPB.

Latent regression analyses were conducted to estimate the multiple correlation between the facets of parenting and IWAH and to explore which facets of attachment and RPB could predict the two facets of IWAH (GSD and GSI) when controlling for the other facets of attachment and RPB. For this purpose, the models which were used in the correlation analyses were modified. In the modified versions of the models, the two facets of IWAH were regressed on the variables representing nRPB, pRPB, and attachment (see Figures 4, 5). The regression models showed the same model fit as the corresponding correlation models. Additionally, one regression analysis per sample was calculated, in which all positive and negative facets were included as predictors (“Combined Model”).

#### Positive model, German sample

All estimated parameters of the positive regression model in the German sample are displayed in Supplementary Table 10. Altogether, the variables representing pRPB and attachment explained 4.1% of the variance in the latent variable GSD as well as in the latent variable GSI (*R*^2^=0.041, *R* = 0.20, *p* < 0.001 for both variables). The variables representing pRPB and attachment were highly correlated: *r*_min_=*r*_*PoP*, *Trs*_=0.845, *r*_max_=*r*_*WrS*, *Lve*_=0.996. Therefore, despite the previously observed positive correlations between both facets of IWAH and all facets of pRPB and attachment, only one of the regression coefficients was significant (GSD is regressed on Communication) and some of the non-significant regression coefficients were even negative.

To avoid the problem of multicollinearity, one additional regression model was constructed for each facet of pRPB and attachment. In each of these models, except for the two regression coefficients between one chosen facet of pRPB or attachment and both facets of IWAH, all regression coefficients were set to zero. Testing these models revealed that without controlling for the other facets, the estimated standardized regression coefficients of all facets lay between β=0.110 (GSI is regressed on Trust) and β=0.178 (GSD is regressed on Communication), explaining between 1.2 and 3.2% of the variance in the two facets of IWAH. In these models, all regression coefficients were significant.

#### Positive model, English sample

All estimated parameters for the data from the English sample are displayed in Supplementary Table 11. Altogether, the variables representing pRPB and attachment could explain about 7% of the observed variance in the facet GSD (*R*^2^=0.067, *R* = 0.26, *p* < 0.001), and about 5% of the variance in the facet GSI (*R*^2^=0.051, *R* = 0.23, *p* < 0.001). In the English sample, the variables representing pRPB and attachment were highly correlated too: *r*_min_=*r*_*PoP*, *Trs*_=0.838, *r*_max_=*r*_*WrS*, *Lve*_=1.011. Thus, the problem of multicollinearity was present in the English sample as well. Therefore, additional models were examined, in which the influence of one facet at a time was tested, without controlling for the other facets of pRPB and attachment. This procedure was already described for the tests in the German sample and will therefore not be repeated here. In the English sample, the estimated standardized regression coefficients of all facets were significant and lay between β=0.122 (GSD is regressed on Love) and β=0.180 (GSD is regressed on Communication), explaining between 1.5 and 3.3% of the variance in the two facets of IWAH.

#### Negative model, German sample

As the negative model allowing for all correlations was rejected, no further regression analysis was conducted for this model with the data from the German sample.

#### Negative model, English sample

All estimated parameters of the model can be found in Supplementary Table 12. Collectively, the facets of nRPB could explain 4.4% of the variance in the facet GSD (*R*^2^=0.044, *R* = 0.21, *p* < 0.001), and 3.4% of the observed variance in GSI (*R*^2^=0.034, *R* = 0.18, *p* < 0.001). There were strong correlations between the facets of nRPB (ranging from *r*_*Ind*, *Ovr*_=0.648 to *r*_*Ind*, *Abs*_=0.844).

Due to the strong correlations, the estimated regression coefficients were confounded with suppression effects. Therefore, the estimated regression coefficients were difficult to interpret. The three significant correlations in the preceding correlation analysis were the correlations between GSD and Abuse, between GSD and Over-Control, and between GSI and Over-Control. Therefore, two additional models were examined, one of which only allowed for regression coefficients between Abuse and the two facets of IWAH, and one of which only allowed for regression coefficients between Over-Control and the two facets of IWAH. When not controlling for the other facets of nRPB, the regression coefficient between Abuse and GSD was significant (β_GSD∼Abs_=0.145, *p* < 0.001), with Abuse explaining 2.1% of the observed variance in GSD. Additionally, the coefficients between Over-Control and GSD (β_GSD∼Ovr_=0.157, *p* < 0.001) and between Over-Control and GSI (β_GSI∼Ovr_=0.084, *p* = 0.008) were significant. The facet Over-Control explained 2.5% of the variance in GSD, and 0.7% of the variance in GSI.

#### Combined model, German sample

The combined model including all positive and all negative facets had an acceptable fit: χ^2^(123) = 474.818, *p* < 0.001; *SRMR* = 0.038; *CFI* = 0.987; *RMSEA* = 0.045,90%*CI*[0.041,0.050]. All estimated parameters of the model can be found in Supplementary Table 13. Collectively, all facets of nRPB, pRPB, and attachment could explain 5.7% of the variance in the facet GSD (*R*^2^=0.057, *R* = 0.24, *p* < 0.001), and 9.2% of the observed variance in GSI (*R*^2^=0.092, *R* = 0.30, *p* < 0.001). Just as in the positive model, there were strong correlations between the variables representing positive parenting and attachment (*r*_min_=*r*_*Lve*, *Cmm*_=0.845). The negative facets were, too, highly correlated: *r*_min_=*r*_*Ind*, *Ovr*_=0.717, *r*_max_=*r*_*Abs*, *Ovr*_=0.917. The correlations between all positive and negative facets ranged between *r*_min_=*r*_*PoP*, *Ovr*_ = -0.352 and *r*_max_=*r*_*Lve*, *Ind*_ = -0.778.

Just as in the preceding analyses with separate models for positive and negative facets, suppression effects resulted in regression coefficients which could not be easily interpreted: non-significant coefficients with some negative and some positive signs for both positive and negative facets. Testing models in which GSD and GSI were regressed on single facets without controlling for the others led to similar results as the preceding analyses with separate models for positive and negative facets: Positive and significant regression coefficients of similar sizes (as in the analyses with separate models) for all positive facets, and non-significant regression coefficients for all negative facets.

#### Combined model, English sample

The combined model including all positive and all negative facets fit the data worse than the separate models: χ^2^(123) = 689.302, *p* < 0.001; *SRMR* = 0.054; *CFI* = 0.980; *RMSEA* = 0.057, 90%*CI*[0.053,0.061]. All estimated parameters of the model can be found in Supplementary Table 14. Collectively, all facets of nRPB, pRPB, and attachment could explain 11.4% of the variance in the facet GSD (*R*^2^=0.114, *R* = 0.34, *p* < 0.001), and 8.1% of the observed variance in GSI (*R*^2^=0.081, *R* = 0.28, *p* < 0.001). Just as in the German sample, there were strong correlations between the variables representing positive parenting and attachment (*r*_min_=*r*_*Lve*, *Cmm*_=0.841) and the negative facets were highly correlated as well: *r*_min_=*r*_*Ind*, *Ovr*_=0.638, *r*_max_=*r*_*Ind*, *Abs*_=0.844. The correlations between all positive and negative facets ranged between *r*_min_=*r*_*Cmm*, *Abs*_ = -0.339 and *r*_max_=*r*_*Lve*, *Ind*_ = -0.699.

As in the previous analyses, suppression effects resulted in regression coefficients which could not be easily interpreted. In the English sample, the regression coefficients of almost all positive facets were non-significant, while those of the negative facets were significant and had different signs. However, testing models in which GSD and GSI were regressed on single facets without controlling for the others led to similar results as the preceding analyses with separate models for positive and negative facets: Positive and significant regression coefficients of similar sizes (as in the analyses with separate models) for all positive facets, significant positive regression coefficients of similar sizes for β_*GSD∼Ovr*_, β_*GSD∼Ovr*_, and β_*GSD∼Ovr*_, and non-significance of all other regression coefficients of negative facets.

#### Ordered probit regression

In addition to the previously described regression analyses, an ordered probit regression analysis was conducted in which the level of agreement to the statement “I see myself as a global citizen” was regressed on the level of agreement to the two statements “I see my mother as a global citizen” and “I see my father as a global citizen”. The levels of agreement to all three statements ranged from 1 (“Strongly disagree”) to 4 (“Strongly agree”). The regression was calculated with the sem() function included in the R package “lavaan” (Rosseel, 2012) and the diagonally weighted least squares (DWLS) estimator (Muthén, 1998). Allowing for a correlation between the two predictors, in the German sample, 33.4% of the variance in the statement “I see myself as a global citizen” could be explained by the other two statements. In the English sample, 25.6% of the variance in the first statement could be explained by the statements concerning participants’ parents.

## Discussion

The aim of this study was to analyze whether there is a relationship between people’s upbringing and their IWAH. More precisely, the relationship between people’s pRPB, attachment, nRPB, and IWAH was analyzed in a German and an English sample. Additionally, the study aimed to explore whether any facets of attachment, nRPB, or pRPB could significantly predict IWAH when controlling for the other facets in a latent regression. In the German as well as in the English sample, the facets of IWAH were found to correlate weakly positively with different facets of attachment (Communication and Trust) and pRPB (Warmth/Support, Positive Parenting, and Love). Regression analyses revealed that between 4.1 and 6.7% of the variance in both facets of IWAH could be explained by these facets of attachment and pRPB. In the English sample, surprisingly, significant positive correlations were found between two facets of nRPB (Over-Control and Abuse) and IWAH. The facet Over-Control, which had the highest correlation with one of the facets of IWAH, could explain 2.5% of the observed variance in GSD when not controlling for the other facets of nRPB.

### Interpretation and discussion of findings

The first hypothesis that was tested in the present study (Hypothesis 1) was that there is a positive correlation between each facet of positive parenting behavior and both facets of IWAH, as well as between each facet of attachment and both facets of IWAH. Analyzing the German sample, this hypothesis was confirmed. In the model which included correlations between all facets of attachment, pRPB and IWAH, all relevant correlations were significant and ranged from *r* = 0.097 (GSI and Trust) to *r* = 0.180 (GSD and Communication). Overall, the facet “Positive Parenting” correlated most strongly with the two facets of IWAH (*r*_*GSD*, *PoP*_=0.173, *r*_*GSI*, *PoP*_=0.176), while the facet “Trust” correlated most weakly with them (*r*_*GSD*.*Trs*_=0.117, *r*_*GSI*, *Trs*_=0.097). Altogether, in the corresponding regression model, all facets of attachment and pRPB explained 4.1% of the observed variance in GSD, just as in GSI. Analyzing the English sample, Hypothesis 1 was accepted as well. All estimated correlations were significant, positive, and ranged from *r* = 0.108 (GSD and Love) to *r* = 0.193 (GSI and Positive Parenting). Just as in the German sample, the facet “Positive Parenting” correlated most strongly with the facet GSD (*r*_*GSD*, *PoP*_=0.180, *r*_*GSI*, *PoP*_=0.193), while the facet which correlated most weakly with IWAH was “Trust” (*r*_*GSD*.*Trs*_=0.119, *r*_*GSI*, *Trs*_=0.115). Altogether, in the corresponding regression model, all facets of attachment and pRPB explained 6.7% of the observed variance in GSD and 5.1% of the observed variance in GSI.

An unpublished study by McFarland et al. (2013) found no relationship between IWAH and people’s memories of their parents’ affection, support, and care (p. 196). Contrarily, the present study found weak correlations between IWAH and the facets of pRPB and attachment. In a latent regression analysis, those facets could explain 4.1 to 6.7% of the variance in the facet GSD, which is considerably more than one would expect in the face of the results reported by McFarland et al. (2013). As the respective study was not published, it is difficult to identify reasons for the differing results. However, another unpublished study by Hamer (2017) with an adult sample from Poland found that IWAH correlated significantly but weakly with parents giving autonomy and acceptance to their child, as well as with a secure attachment style (McFarland et al., 2019, p. 160). The results of the present study match these findings. However, when interpreting these results, it should also be kept in mind that the correlations could be significant due to large sample sizes and that most of them are very weak.

As openness to experience (Hamer et al., 2019) as well as multicultural experiences (Sparkman and Hamer, 2020) have been found to predict IWAH and as they are also likely to show positive correlations with pRPB and attachment, they might be mediators of the relationship between pRPB/attachment and IWAH. Positive relationships in childhood and youth might increase openness to new relationships, including relationships to people from different cultures, which might in turn increase IWAH. Other possible moderators include perspective taking and empathetic concern, which could both be increased by positive parenting and which also have been found to correlate positively with IWAH (McFarland et al., 2012).

The second hypothesis that was tested in the present study (Hypothesis 2) was that there is a negative correlation between each facet of negative parenting behavior and both facets of IWAH. For the German sample, this hypothesis was rejected as the model allowing for all correlations did not fit the data significantly better than the model in which all relevant correlations were set to zero. Analyzing the English sample, the model allowing for all correlations fit the data significantly better. However, Hypothesis 2 also needed to be rejected for the English sample, as firstly, half of the correlations were not significant, and secondly, contrary to Hypothesis 2, all significant correlations between the facets of nRPB and IWAH were positive. The three significant positive correlations were the correlation between GSD and abuse (*r* = 0.153, *p* < 0.001), the correlation between GSD and over-control (*r* = 0.151, *p* < 0.001), and the one between GSI and over-control (*r* = 0.088, *p* = 0.011). When controlling the *FDR*, all three correlations remained significant. This result was surprising, as the positive correlations between GSD and abuse as well as GSD and over-control were as high as most correlations in the positive model. In the corresponding negative regression model, the facet abuse explained 2.1% and the facet over-control explained 2.5% of the observed variance in GSD when not controlling for the other facets of nRPB.

So far, the present study is the first to discover a correlation between nRPB and IWAH. The unpublished study by McFarland et al. (2013) already mentioned when discussing Hypothesis 1 found IWAH to be unrelated to remembered punitive and spoiling parenting behavior (p. 196). Besides, Hamer and McFarland (2018, June) found IWAH to be unrelated to harsh and strict, as well as to unaffectionate socialization (McFarland et al., 2019). As no correlations between IWAH and nRPB were found in the German sample and as half of the correlations in the English sample were non-significant, the present study mostly confirmed the results of the two preceding studies. Nevertheless, it is unclear, why the present study found significant, albeit small positive correlations between GSD and abuse as well as GSD and GSI and over-control in the English sample. It is worth noting that these significant correlations could also trace back to a large sample size and that variables which have not been discovered yet might moderate the strength of these correlations. Future studies could try to further investigate these relationships.

To summarize, there are weak indications that pRPB and attachment could contribute to the development of IWAH, while there are no indications that nRPB could prevent it. Considering implications, this result is very comforting, as it shows that experiencing positive parenting behavior might slightly enhance IWAH while experiencing negative parenting behavior probably does not inhibit IWAH.

Because most correlations were very small, we conducted an exploratory search for alternative parent-related antecedents of individuals’ IWAH. In an ordered probit regression, two brief statements on parents’ cosmopolitanism explained about one third of the variance in the statement “I see myself as a global citizen” (25.6% in the English and 33.4% in the German sample). This result indicates that parents’ attitudes or behavior specifically related to IWAH could have a greater impact on people’s IWAH than more unspecific parenting behavior.

### Limitations

Although the present study and its analyzed models were planned with care, some limitations should be considered when interpreting the results, eight of which are discussed in this section.

Firstly, all participants came from Germany or England, two Western, educated, industrialized, rich, and democratic (WEIRD) nations (Henrich et al., 2010; Muthukrishna et al., 2020). The two samples analyzed in the study are representative for their respective nations, but for those nations only. Therefore, the study does not allow to draw inferences about other nations, unless subsequent studies with data from other countries are conducted to replicate the results. The data collection for this study was part of a larger project with different research questions. Some of the questions had to do with the European Union, which is our reason for having added the category “Europeans” to the IWAH scale and which is also the reason for having chosen a country which is part of the EU for our first (German) sample. We were also interested in questions concerning Brexit, which is the reason for having chosen the English sample. However, we do not have any reason for having chosen Germany rather than another European country for our first sample other than our own ability to understand and check German translations of the questionnaires and the survey.

Secondly, the analyzed data might contain a slight bias toward people being religious. As one of the quality check items was included in a questionnaire which was only relevant for people who reported to remember a significant religious or spiritual event, more non-religious than religious participants failed to answer this quality check item correctly and were therefore excluded from the study if they took part in the study from June 24 to July 9, 2021. However, it is unlikely that a slight bias toward people being more religious has any influence on the relationship between RPB, attachment, and IWAH, as religious faith has been found to be either unrelated or only weakly negatively related to IWAH (McFarland et al., 2019).

Thirdly, the models which were used to analyze the correlations and predictive relationships between the variables were slightly modified in the CFA. All changes from the initial models were driven by the present data structure. Although it was ensured that all modifications were in line with prior theoretical assumptions, necessary modifications might differ in other samples. On that account, the SEM analyzed in the present study should be replicated in other samples, as their generalizability still needs to be tested.

Fourthly, the Measure of Parental Style (MOPS; Parker et al., 1997), which was used to obtain items measuring RPB, was originally designed to measure parenting styles. As the definition of parenting styles and parenting behavior differs slightly, this choice deserves explanation. The MOPS was chosen for measuring nRPB because it is one of the only questionnaires covering negative parenting behavior, which is validated for English as well as German samples, and which has an adequate length. Moreover, the items in the MOPS are fairly concrete (e.g., “My mother/father left me on my own a lot”). Therefore, they are in line with the definition of RPB used in the study.

Fifthly, all analyses in the present study relied on self-reports. It is unclear whether other methods like observer ratings or interviews would have led to the same results. As GSD and GSI as well as parenting behavior are complex constructs, directly talking to people and allowing them to explain what they associate with the questionnaires’ items could yield valuable new insights into the topic.

Sixthly, it is beyond the scope of this study to confirm causal relationships between RPB, attachment, and IWAH. Although the regression models fit the data well and although several regression coefficients were significant, the study design does not allow for causal conclusions on the relationship between RPB, attachment, and IWAH to be drawn. Future studies could try to investigate whether other variables might moderate the observed relationships between the variables. However, the present study indicates that the idea of parenting behavior influencing IWAH should not be rejected prematurely.

Seventhly, we did not randomize the order of the different questionnaires presented in the survey across participants. The IWAH scale was placed right at the beginning of the survey so that answers could not be influenced by preceding questionnaires. The questionnaires concerning parenting and attachment were placed as far away from the IWAH scale as possible for participants not to give similar answers by habit. Although we assume that neither order effects, nor the contents of other questionnaires presented in the survey biased the answers to the questions concerning parenting and attachment, we cannot fully exclude this possibility.

And eighthly, the selection of the facets representing RPB as well as attachment lacks theoretical justification. When planning the study, our idea was to investigate in a general way, whether positive parenting has an overall positive relationship with IWAH, and whether negative parenting has an overall negative relationship with IWAH. Thus, the concrete facets were not the main focus of our work. We chose measures for parenting behavior and attachment pragmatically, focusing on availability, validity in Germany as well as in England, and reliability. For instance, we chose to measure attachment with modified items from the Inventory of Parent and Peer Attachment (IPPA; Armsden and Greenberg, 1987) because the scale was available, the facets of interest were already validated in German as well as English samples, and because we had already used the scale in other projects, where it had turned out to be a reliable measure (Bohn et al., 2020). The items measuring attachment were close to our working definition of parenting behavior while still adding new aspects. Therefore, the two concepts positive parenting and attachment as used in our study show some overlap and are not clearly distinct. Undoubtedly, there are more popular ways to classify attachment, for instance classifying secure, avoidant, anxious-ambivalent, and disorganized-disoriented attachment according to Ainsworth et al. (1978/2015). Our study does not allow for any conclusions concerning IWAH and attachment styles as they are commonly defined.

### Implications and future directions

Identification with all humanity has been shown to predict several positive behavioral outcomes, like the desire for global knowledge, a willingness to contribute to humanitarian relief, forgiving former enemies of their war crimes (Hamer et al., 2017, 2018), volunteering (Stürmer et al., 2016; Faulkner, 2018), and cooperative health behavior during the COVID-19 pandemic (Barragan et al., 2021; Marchlewska et al., 2022; Sparkman et al., 2022). In addition to that, people’s cosmopolitan orientation, which is similar to IWAH, has been shown to predict pro-environmental behaviors above and beyond other factors like a pro-environmental worldview, pro-environmental motivations and beliefs (Leung et al., 2015).

Trying to investigate the origins of IWAH, the present study found that pRPB and nRPB might have a small predictive impact on GSD and GSI. This finding leaves room for several questions future studies could revisit: What are other possible sources of IWAH? Do parents’ attitudes or behavior more specifically related to IWAH have a greater impact on people’s IWAH? Are there relevant mediators for the observed relationship between pRPB and IWAH? And under which circumstances does a person’s GSI transform into concrete actions aimed at helping other human beings who are in need? There is a great urgency to deal with current global challenges like the climate change on our shared planet and global migration. Trying to find answers to the questions above is not only interesting but could also yield effective approaches to tackle these challenges.

As the present study was one of the first to analyze the relationship between RPB, attachment, and IWAH, there are some aspects it could not cover. Further research is needed to test the generalizability of the study’s findings. Replications in other countries could help to test whether similar results can be found in other parts of the world. It is particularly interesting to find out whether the observed positive correlations between Abuse and GSD as well as Over-Control and GSD and GSI can be replicated in other samples. Subsequent studies could also try to investigate whether other variables might moderate the observed relationships between pRPB, attachment, and IWAH. Additionally, further research could explore other possible sources of IWAH and other possible ways to promote it. For example, future studies could focus on aspects of parenting that are specifically related to IWAH. When doing so, different research methods like interviews or observer ratings could yield new insights into the topic.

Now can we raise citizens of the world? The present study indicates that we might. Comforting our children when they are in trouble, showing them love and affection, asking them about their hobbies and interests and giving them a “compliment, hug, or a tap on the shoulder” (Meunier and Roskam, 2007, p. 116) every now and then will surely cause no harm.

## Data availability statement

The raw data supporting the conclusions of this article will be made available by the authors, without undue reservation.

## Ethics statement

Ethical review and approval was not required for the study on human participants in accordance with the local legislation and institutional requirements. The patients/participants provided their written informed consent to participate in this study.

## Author contributions

MH, FT, and ME contributed to the conception of the study. MH wrote the manuscript, created the tables and figures, and conducted all data analyses. FT defined the inclusion criteria, organized/conducted the survey, and communicated with LimeSurvey and the respondi AG as well as with other researchers. ME provided statistical advice and monitored the whole research process. FT and ME participated in the revision of the article. All authors have read and approved the final version.

## Footnotes

1 The second quality check item had only been presented to a subsample and would thus have biased the exclusion, while the third quality check item was positioned after the last questionnaire relevant to the present study.

## Abbreviations

IWAH: Identification With All Humanity
RWA: right-wing authoritarianism
SDO: social dominance orientation
GSD: global self-definition
GSI: global self-investment
RPB: remembered parenting behavior
pRPB: positive remembered parenting behavior
nRPB: negative remembered parenting behavior
CFA: confirmatory factor analysis/analyses
SEM: structural equation modeling/structural equation model(s)
SRMR: Standardized Root Mean Square Residual(s)
CFI: Comparative Fit Index/Indices
RMSEA: Root Mean Square Error(s) of Approximation
BIC: Bayesian Information Criterion/Criteria
FDR: False Discovery Rate.
