# Supplementary material for: Perceived parenting and identification with all humanity: Insights from England and Germany
Source: Front Psychol. 2022 Aug 3;13:924562. doi: 10.3389/fpsyg.2022.924562 (PMC9381749; doi:10.3389/fpsyg.2022.924562)
Supplement: Supplementary file 1 [file Data_Sheet_1.pdf]

Supplementary Material

1 Tables

Table S1

Sociodemographic Characteristics of the Total, the German, and the English Sample

Variable		Total sample (n = 3056)	German sample (n = 1517)	English sample (n = 1539)
Age	*M (SD)*	46.47 (15.51)	46.61 (15.51)	46.33 (15.51)
Gender				
Female	*n (%)*	1414 (50.34%)	719 (50.56%)	695 (50.11%)
Male	*n (%)*	1377 (49.02%)	700 (49.23%)	677 (48.81%)
Non-binary	*n (%)*	16 (0.56%)	3 (0.21%)	13 (0.94%)
I would rather not say	*n (%)*	2 (0.07%)	0 (0%)	2 (0.14%)
Total monthly household income				
£0 to £1649 (0€ to 1999€)	*n (%)*	711 (25.31%)	401 (28.20%)	310 (22.35%)
£1650 to £2999 (2000€ to 3999€)	*n (%)*	1134 (40.37%)	499 35.09%)	635 (45.78%)
> £3000 (> 4000€)	*n (%)*	964 (34.32%)	522 (36.71%)	442 (31.87%)
Highest level of education[1]				
Children's Centre	*n (%)*	6 (0.21%)	2 (0.14%)	4 (0.28%)
Primary school	*n (%)*	12 (0.42%)	9 (0.62%)	3 (0.21%)
Secondary school	*n (%)*	602 (21.01%)	359 (24.93%)	243 (17.05%)
Higher Level Diploma	*n (%)*	681 (23.77%)	351 (24.38%)	330 (23.16%)
Higher National Certificate	*n (%)*	434 (15.15%)	327 (22.71%)	107 (7.51%)
Higher National Diploma	*n (%)*	136 (4.75%)	37 (2.57%)	99 (6.95%)
Bachelor's degree	*n (%)*	550 (19.20%)	172 (11.94%)	378 (26.52%)
Master's degree	*n (%)*	376 (13.12%)	157 (10.90%)	219 (15.37%)
Doctorate	*n (%)*	54 (1.88%)	20 (1.39%)	34 (2.39%)
Other	*n (%)*	14 (0.49%)	6 (0.42%)	8 (0.56%)

Variable		Total sample (n = 3056)	German sample (n = 1517)	English sample (n = 1539)
Members of a religious congregation or community	*n (%)*	793 (25.95%)	505 (33.29%)	288 (18.71%)
Grown up bilingually	*n (%)*	319 (10.44%)	151 (9.95%)	168 (10.92%)
Number of languages spoken fluently	*M (SD)*	1.46 (.76)	1.61 (.79)	1.31 (.69)
Having been to another country	*n (%)*	2644 (86.52%)	1331 (87.74%)	1313 (85.31%)
Having lived in another country	*n (%)*	724 (23.69%)	259 (17.07%)	465 (30.21%)
Nationalities[2]				
German	*n (%)*	1413 (46.36%)	1407 (94.81%)	6 (0.38%)
English	*n (%)*	762 (25.00%)	2 (0.13%)	760 (48.59%)
British	*n (%)*	522 (17.13%)	1 (0.07%)	521 (33.31%)
Scottish	*n (%)*	40 (1.31%)	0 (0%)	40 (2.56%)
Italian	*n (%)*	32 (1.05%)	10 (0.67%)	22 (1.41%)
Irish	*n (%)*	21 (0.69%)	0 (0%)	21 (1.34%)
Polish	*n (%)*	21 (0.69%)	6 (0.40%)	15 (0.96%)
Welsh / Cymro / Cymraes	*n (%)*	20 (0.66%)	0 (0%)	20 (1.28%)
Indian	*n (%)*	15 (0.49%)	0 (0%)	15 (0.96%)
French	*n (%)*	10 (0.33%)	3 (0.20%)	7 (0.45%)
Romanian	*n (%)*	10 (0.33%)	2 (0.13%)	8 (0.51%)
Portuguese	*n (%)*	10 (0.33%)	3 (0.20%)	7 (0.45%)
Others	*n (%)*	172 (5.64%)	50 (3.37%)	122 (7.80%)

Notes. Statistics in the table refer to the data from the participants included in the final samples. Percentages always refer to the total number of people for whom the respective information was given. [1] Levels of education were slightly different for the English and the German sample due to national differences in the education system. The exact levels of education presented to the participants can be found in Table S3. [2] Only those nationalities which at least 10 participants in the total sample reported to have are listed here.

Table S2

People for Whom Participants Assessed RPB and Attachment

		German sample (n = 1517)	English sample (n = 1539)
Items about the “mother” were answered for the participants’:			
Biological mother	*n (%)*	1485 (97.89%)	1510 (98.12%)
Stepmother	*n (%)*	9 (0.59%)	8 (0.52%)
Second mother	*n (%)*	3 (0.20%)	0 (0%)
Foster mother	*n (%)*	5 (0.33%)	1 (0.06%)
Adoptive mother	*n (%)*	3 (0.20%)	11 (0.71%)
Grandmother	*n (%)*	7 (0.46%)	3 (0.19%)
Aunt	*n (%)*	2 (0.13%)	1 (0.06%)
Someone else	*n (%)*	3 (0.20%)	4 (0.26%)
Items about the “father” were answered for the participants’:			
Biological father	*n (%)*	1415 (93.28%)	1459 (94.80%)
Stepfather	*n (%)*	55 (3.63%)	39 (2.53%)
Second father	*n (%)*	1 (0.07%)	2 (0.13%)
Foster father	*n (%)*	5 (0.33%)	0 (0%)
Adoptive father	*n (%)*	16 (1.05%)	15 (0.97%)
Grandfather	*n (%)*	3 (0.20%)	4 (0.26%)
Uncle	*n (%)*	1 (0.07%)	2 (0.13%)
Biological mother	*n (%)*	0 (0%)	2 (0.13%)
Stepmother	*n (%)*	1 (0.07%)	0 (0%)
Grandmother	*n (%)*	4 (0.26%)	0 (0%)
Aunt	*n (%)*	2 (0.13%)	1 (0.06%)
Another relative	*n (%)*	0 (0%)	1 (0.06%)
Someone else	*n (%)*	14 (0.92%)	14 (0.91%)

Notes. One participant from the English sample reported to have answered the items concerning her mother for her biological father and all items about her father for her biological mother. For this participant, the given answers were switched in the analyses.

Table S3

Levels of Education for the English and the German Sample

Group	English sample	German sample
0	• Children's Centre (including Sure Start centre)<br>• Registered Childminder<br>• Day nursery<br>• Reception or Nursery class in a school	• Krippe<br>• Kindergarten<br>• Vorschule/Vorklasse
1	• Primary school<br>• Adult literacy, numeracy and language	• Grundschule<br>• Integrierte Gesamtschule (bis Klasse 4)<br>• Freie Waldorfschule (bis Klasse 4)<br>• Förderschule (bis Klasse 4)
2	• Secondary school<br>• GCSE (grades 3 to 1 / D to G)<br>• Foundation Level Diploma<br>• NVQ Level 1	• Allgemeiner Schulabschluss Sekundarstufe I / Mittlere Reife (Förderschule bis Klasse 10,<br>• Hauptschule, Abendhauptschule, Realschule, Abendrealschule, Integrierte Gesamtschule bis Klasse 10, Freie Waldorfschule bis Klasse 10, Gymnasium bis Klasse 9/10, Berufsfachschule, Berufsschule)<br>• Berufsaufbauschule, Berufseinstiegsklasse<br>• berufliche Grundbildung (Berufsfachschule)<br>• Berufsvorbereitung (Berufsfachschule)<br>• Berufsgrundbildungsjahr<br>• Praktikum vor der Erzieher*innenausbildung<br>• Berufsschule ohne Ausbildungsvertrag
3	• Higher Level Diploma<br>• Advanced Level Diploma<br>• NVQ Level 2-3<br>• Apprenticeship (Intermediate, Advanced)<br>• GCSE (grades 9 to 4 / A* to C)<br>• Access to HE Diploma	• Abitur / Allgemeiner Schulabschluss Sekundarstufe II / Allgemeine Hochschulreife / Studienberechtigung / Abschluss der gymnasialen Oberstufe (Förderschule, Freie Waldorfschule, oder Integrierte Gesamtschule bis Klasse 13, Gymnasium bis Klasse 12/13)<br>• Fachabitur (Fachgymnasium, Berufsfachschule)<br>• Berufliche Erstausbildung (Berufskolleg, Berufsschule, Berufsfachschule, zweijährige<br>• Fachoberschule ohne Berufsausbildung)<br>• Kurzausbildung in Gesundheits- und Sozialberufen (Berufsfachschule, Schule des Gesundheitswesens, Kurzprogramme)<br>• Beamtenausbildung (mittlerer Dienst)<br>• Vermittlung beruflicher Kenntnisse für Abiturient*innen (Berufsfachschule)

4	• NVQ Level 4<br>• Higher National Certificate (HNC)	• Berufliche Ausbildung(en) und Studienberechtigung (Berufsschule, Fachgymnasium, Berufsfachschule)<br>• Berufliche Zweitausbildung (nach vorherigem Erwerb einer Studienberechtigung)<br>• Berufliche Drittausbildung (nach vorherigem Erwerb einer Studienberechtigung und einer<br>• beruflichen Ausbildung)<br>• Zwei- oder dreijähriger Bildungsgang in Gesundheits- oder Sozialberuf (Berufsfachschule, Schule des Gesundheitswesens, Fachschule)<br>• Abendschulabschluss Sekundarstufe II<br>• Einjährige Fachoberschule mit vorheriger Berufsausbildung<br>• Berufsoberschule<br>• Technische Oberschule<br>• Berufliche Umschulung (Berufsschule, dual)
5	• NVQ Level 5<br>• Higher National Diploma (HND)<br>• Diploma of Higher Education<br>• Foundation degree	• Meisterausbildung (bis zu drei Jahre)
6	• Bachelor's degree	• Meisterausbildung (drei Jahre oder länger)<br>• Fachschulausbildung (Erzieher*in, Techniker*in, Fachwirt*in, Betriebswirtschaft, Informationstechnik, Hauswirtschaft)<br>• Fachakademieabschluss (Bayern)<br>• Erzieher*innenausbildung (Berufsfachschule)<br>• Diplom (Berufsakademie, Verwaltungsfachhochschule, Fachhochschule)<br>• Bachelor
7	• Master's degree<br>• Post-graduate diploma/certificate	• Master<br>• Diplom (Universität)<br>• Aufbau-/Ergänzungsstudium (Universität)
8	• Doctorate	• Promotion
9	• Other	• Anderer

Table S4

Values Entered in the Power Analysis for the Positive Model

Parameter	Value
Factor loading	
Warmth/Support measured by ZKE Items 1 and 4	0.64
Warmth/Support measured by ZKE Items 2 and 3	0.61
Positive Parenting measured by EPEP Items 1 and 4	0.73
Positive Parenting measured by EPEP Items 2 and 3	0.68
Love measured by LVE Items 1 and 2	0.68
Love measured by LVE Items 3 and 4	0.68
Trust measured by IPPA Items 13 and 21	0.66
Trust measured by IPPA Items 1 and 4	0.7
Communication measured by IPPA Items 15 and 19	0.67
Communication measured by IPPA Items 16 and 24	0.7
GSD measured by IWAH Items 1 and 4	0.65
GSD measured by IWAH Items 2 and 3	0.67
GSI measured by IWAH Items 6 and 7	0.67
GSI measured by IWAH Items 8 and 9	0.69
Covariance	
Variance of Warmth/Support covaries with variance of GSD	0.2
Variance of Positive Parenting covaries with variance of GSD	0.2
Variance of Love covaries with variance of GSD	0.2
Variance of Trust covaries with variance of GSD	0.2
Variance of Communication covaries with variance of GSD	0.2
Variance of Warmth/Support covaries with variance of GSI	0.2
Variance of Positive Parenting covaries with variance of GSI	0.2
Variance of Love covaries with variance of GSI	0.2
Variance of Trust covaries with variance of GSI	0.2
Variance of Communication covaries with variance of GSI	0.2
Variance of Warmth/Support covaries with variance of Positive Parenting	0.5

Variance of Warmth/Support covaries with variance of Love	0.5
Variance of Warmth/Support covaries with variance of Trust	0.3
Variance of Warmth/Support covaries with variance of Communication	0.3
Variance of Positive Parenting covaries with variance of Love	0.5
Variance of Positive Parenting covaries with variance of Trust	0.24
Variance of Positive Parenting covaries with variance of Communication	0.37
Variance of Love covaries with variance of Trust	0.5
Variance of Love covaries with variance of Communication	0.5
Variance of Trust covaries with variance of Communication	0.76
Variance of GSD covaries with variance of GSI	0.77
Residual variance	
Residual variance of ZKE Items 1 and 4	0.5904
Residual variance of ZKE Items 2 and 3	0.6279
Residual variance of EPEP Items 1 and 4	0.4671
Residual variance of EPEP Items 2 and 3	0.5376
Residual variance of LVE Items 1 and 2	0.5376
Residual variance of LVE Items 3 and 4	0.5376
Residual variance of IPPA Items 13 and 21	0.5644
Residual variance of IPPA Items 1 and 4	0.51
Residual variance of IPPA Items 15 and 19	0.5511
Residual variance of IPPA Items 16 and 24	0.51
Residual variance of IWAH Items 1 and 4	0.5775
Residual variance of IWAH Items 2 and 3	0.5511
Residual variance of IWAH Items 6 and 7	0.5511
Residual variance of IWAH Items 8 and 9	0.5239

Notes. Variances of all latent variables were fixed and are therefore not reported here. GSD = Global self-definition, GSI = Global self-investment. The following abbreviations were used for the different questionnaires: ZKE = „Zürcher Kurzfragebogen zum Erziehungsverhalten", EPEP = "Evaluation des Pratiques Educatives Parentales", LVE = items measuring parental love, IPPA = "Inventory of Parent and Peer Attachment", IWAH = "Identification With All Humanity scale".

Table S5

Values Entered in the Power Analysis for the Negative Model

Parameter	Value
Factor loading	
Indifference measured by MOPS Items 5 and 13	0.87
Indifference measured by MOPS Items 12 and 8	0.85
Abuse measured by MOPS Items 2 and 14	0.65
Abuse measured by MOPS Items 15 and 7	0.59
Overcontrol measured by MOPS Items 4 and 3	0.55
Overcontrol measured by MOPS Items 1 and 6	0.57
GSD measured by IWAH Items 1 and 4	0.65
GSD measured by IWAH Items 2 and 3	0.67
GSI measured by IWAH Items 6 and 7	0.67
GSI measured by IWAH Items 8 and 9	0.69
Covariance	
Variance of Indifference covaries with variance of GSD	-0.2
Variance of Abuse covaries with variance of GSD	-0.2
Variance of Overcontrol covaries with variance of GSD	-0.2
Variance of Indifference covaries with variance of GSI	-0.2
Variance of Abuse covaries with variance of GSI	-0.2
Variance of Overcontrol covaries with variance of GSI	-0.2
Variance of Indifference covaries with variance of Abuse	0.72
Variance of Indifference covaries with variance of Overcontrol	0.54
Variance of Abuse covaries with variance of Overcontrol	0.61
Variance of GSD covaries with variance of GSI	0.77
Residual variance	
Residual variance of MOPS Items 5 and 13	0.2431
Residual variance of MOPS Items 12 and 8	0.2775
Residual variance of MOPS Items 2 and 14	0.5775
Residual variance of MOPS Items 15 and 7	0.6519

Residual variance of MOPS Items 4 and 3	0.6975
Residual variance of MOPS Items 1 and 6	0.6751
Residual variance of IWAH Items 1 and 4	0.5775
Residual variance of IWAH Items 2 and 3	0.5511
Residual variance of IWAH Items 6 and 7	0.5511
Residual variance of IWAH Items 8 and 9	0.5239

Notes. Variances of all latent variables were fixed and are therefore not reported here. GSD = Global self-definition, GSI = Global self-investment. The following abbreviations were used for the two different questionnaires: MOPS = "Measure of Parental Style", IWAH = "Identification With All Humanity scale".

Table S6

Positive Model Allowing for all Correlations in the German Sample: Estimated Parameters

Parameters	Estimate	*SE*	*z*-value	*p*-value	Std. estimate
Factor loadings					
GSD → IWAH 1 & 4	1				0. 863
GSD → IWAH 2 & 3	1.121	0.029	39.130	< .001***	0. 888
GSI → IWAH 6 & 7	1				0.913
GSI → IWAH 8 & 9	1.013	0.024	42.691	< .001***	0.876
WrS → ZKE 1 & 3	1				0.936
WrS → ZKE 2 & 4	1.003	0.013	80.107	< .001***	0.942
PoP → EPEP 1 & 4	1				0.669
PoP → EPEP 2 & 3	1.411	0.113	12.442	< .001***	0.912
Lve → LVE 1 & 2	1				0.946
Lve → LVE 3 & 4	0.962	0.011	88.501	< .001***	0.950
Cmm → IPPA 15 & 19	1				0.961
Cmm → IPPA 16 & 24	0.978	0.010	100.705	< .001***	0.953
Cmm → IPPA 13 & 21	0.514	0.028	18.096	< .001***	0.530
Cmm → EPEP 1 & 4	0.274	0.052	5.298	< .001***	0.288
Trs → IPPA 13 & 21	1				0.460
Trs → IPPA 1 & 4	2.154	0.145	14.888	< .001***	0.978
Covariances					
GSD, GSI	2.215	0.102	21.629	< .001***	0.774
WrS	0.154	0.037	4.178	< .001***	0.128
PoP	0.150	0.029	5.098	< .001***	0.173
Lve	0.144	0.038	3.751	< .001***	0.115
Cmm	0.245	0.042	5.809	< .001***	0.180
Trs	0.071	0.019	3.671	< .001***	0.117
GSI, WrS	0.236	0.046	5.080	< .001***	0.155
PoP	0.192	0.036	5.277	< .001***	0.176
Lve	0.216	0.048	4.469	< .001***	0.137
Cmm	0.220	0.052	4.225	< .001***	0.128
Trs	0.074	0.024	3.113	.002**	0.097
WrS, PoP	0.445	0.037	12.019	< .001***	0.970
Lve	0.664	0.021	31.486	< .001***	0.996

Parameters	Estimate	*SE*	*z*-value	*p*-value	Std. estimate
Covariances					
WrS, Cmm	0.632	0.021	30.702	< .001***	0.876
Trs	0.294	0.022	13.457	< .001***	0.912
PoP, Lve	0.435	0.036	12.059	< .001***	0.910
Cmm	0.469	0.041	11.387	< .001***	0.907
Trs	0.195	0.021	9.106	< .001***	0.845
Lve, Cmm	0.635	0.021	30.575	< .001***	0.847
Trs	0.309	0.023	13.516	< .001***	0.921
Cmm, Trs	0.312	0.023	13.390	< .001***	0.859
Variances					
GSD	2.268	0.118	19.241	< .001***	1
GSI	3.606	0.155	23.271	< .001***	1
WrS	0.640	0.022	29.388	< .001***	1
PoP	0.329	0.052	6.320	< .001***	1
Lve	0.693	0.023	29.623	< .001***	1
Cmm	0.812	0.025	33.002	< .001***	1
Trs	0.162	0.021	7.775	< .001***	1
IWAH 1 & 4	0.778	0.059	13.236	< .001***	0.256
IWAH 2 & 3	0.765	0.068	11.227	< .001***	0.211
IWAH 6 & 7	0.721	0.075	9.574	< .001***	0.167
IWAH 8 & 9	1.123	0.086	13.088	< .001***	0.233
ZKE 1 & 3	0.090	0.005	17.304	< .001***	0.124
ZKE 2 & 4	0.081	0.005	16.812	< .001***	0.112
EPEP 1 & 4	0.088	0.005	16.854	< .001***	0.119
EPEP 2 & 3	0.132	0.010	12.902	< .001***	0.168
LVE 1 & 2	0.082	0.006	14.576	< .001***	0.106
LVE 3 & 4	0.069	0.007	10.085	< .001***	0.097
IPPA 15 & 19	0.067	0.006	11.335	< .001***	0.076
IPPA 16 & 24	0.079	0.006	13.559	< .001***	0.092
IPPA 13 & 21	0.068	0.004	15.903	< .001***	0.088
IPPA 1 & 4	0.034	0.011	3.221	.001**	0.044

Parameters	Estimate
R^2 (Reliabilities)	
Composite[1]	
Parcels	
GSD	0.869
IWAH 1 & 4	0.744
IWAH 2 & 3	0.789
GSI	0.888
IWAH 6 & 7	0.833
IWAH 8 & 9	0.767
WrS	0.937
ZKE 1 & 3	0.876
ZKE 2 & 4	0.888
PoP	0.922
EPEP 1 & 4	0.881
EPEP 2 & 3	0.832
Lve	0.947
LVE 1 & 2	0.894
LVE 3 & 4	0.903
Cmm	0.954
IPPA 15 & 19	0.924
IPPA 16 & 24	0.908
Trs	0.965
IPPA 13 & 21	0.912
IPPA 1 & 4	0.956

Notes. *N* = 1517. *SE* = Standard error, Std. estimate = Standardized estimate (estimate in the fully standardized model). GSD = Global self-definition, GSI = Global self-investment, WrS = Warmth / Support, PoP = Positive parenting, Lve = Love, Trs = Trust, Cmm = Communication. The following abbreviations were used for the questionnaires: ZKE = „Zürcher Kurzfragebogen zum Erziehungsverhalten", EPEP = "Evaluation des Pratiques Educatives Parentales", LVE = items measuring parental love, IPPA = "Inventory of Parent and Peer Attachment", IWAH = "Identification With All Humanity scale". [1] Composite reliabilities of the factors (McDonald's ω) were calculated with the reliability() function of the R package "semTools" (Jorgensen et al., 2022). For factors with cross-loadings (PoP and Trs), reliabilities were calculated using formula (2) in Raykov and Shrout (2002). *$p < .05$. **$p < .01$. ***$p < .001$.

Table S7

Positive Model Allowing for all Correlations in the English Sample: Estimated Parameters

Parameters	Estimate	*SE*	*z*-value	*p*-value	Std. estimate
Factor loadings					
GSD → IWAH 1 & 4	1				0.798
GSD → IWAH 2 & 3	1.150	0.041	27.789	$< .001^{***}$	0.865
GSI → IWAH 6 & 7	1				0.893
GSI → IWAH 8 & 9	1.039	0.027	38.347	$< .001^{***}$	0.900
WrS → ZKE 1 & 3	1				0.923
WrS → ZKE 2 & 4	1.012	0.013	79.899	$< .001^{***}$	0.940
PoP → EPEP 1 & 4	1				0.651
PoP → EPEP 2 & 3	1.492	0.094	15.948	$< .001^{***}$	0.938
Lve → LVE 1 & 2	1				0.919
Lve → LVE 3 & 4	0.928	0.014	64.525	$< .001^{***}$	0.936
Cmm → IPPA 15 & 19	1				0.965
Cmm → IPPA 16 & 24	0.975	0.010	98.082	$< .001^{***}$	0.954
Cmm → IPPA 13 & 21	0.493	0.030	16.327	$< .001^{***}$	0.515
Cmm → EPEP 1 & 4	0.281	0.038	7.400	$< .001^{***}$	0.297
Trs → IPPA 13 & 21	1				0.469
Trs → IPPA 1 & 4	2.044	0.147	13.938	$< .001^{***}$	0.968
Covariances					
GSD, GSI	1.895	0.095	19.848	$< .001^{***}$	0.692
WrS	0.140	0.035	4.058	$< .001^{***}$	0.127
PoP	0.142	0.026	5.414	$< .001^{***}$	0.180
Lve	0.119	0.034	3.505	$< .001^{***}$	0.108
Cmm	0.234	0.042	5.579	$< .001^{***}$	0.183
Trs	0.069	0.019	3.660	$< .001^{***}$	0.119
GSI, WrS	0.253	0.044	5.793	$< .001^{***}$	0.169
PoP	0.206	0.033	6.151	$< .001^{***}$	0.193
Lve	0.229	0.044	5.188	$< .001^{***}$	0.154
Cmm	0.208	0.050	4.204	$< .001^{***}$	0.120
Trs	0.090	0.024	3.754	$< .001^{***}$	0.115
WrS, PoP	0.418	0.028	14.864	$< .001^{***}$	0.971
Lve	0.608	0.021	28.904	$< .001^{***}$	1.011

Parameters	Estimate	*SE*	*z*-value	*p*-value	Std. estimate
Covariances					
WrS, Cmm	0.616	0.020	30.745	< .001***	0.881
Trs	0.284	0.022	12.628	< .001***	0.904
PoP, Lve	0.400	0.027	14.886	< .001***	0.933
Cmm	0.439	0.031	14.309	< .001***	0.881
Trs	0.188	0.018	10.412	< .001***	0.838
Lve, Cmm	0.587	0.020	29.534	< .001***	0.843
Trs	0.285	0.022	12.688	< .001***	0.911
Cmm, Trs	0.310	0.024	13.072	< .001***	0.851
Variances					
GSD	2.022	0.123	16.459	< .001***	1
GSI	3.713	0.173	21.516	< .001***	1
WrS	0.603	0.022	27.970	< .001***	1
PoP	0.307	0.037	8.319	< .001***	1
Lve	0.600	0.024	25.410	< .001***	1
Cmm	0.810	0.023	34.512	< .001***	1
Trs	0.164	0.022	7.376	< .001***	1
IWAH 1 & 4	1.156	0.076	15.143	< .001***	0.364
IWAH 2 & 3	0.904	0.097	9.307	< .001***	0.253
IWAH 6 & 7	0.944	0.097	9.782	< .001***	0.203
IWAH 8 & 9	0.940	0.097	9.730	< .001***	0.190
ZKE 1 & 3	0.105	0.006	18.450	< .001***	0.148
ZKE 2 & 4	0.082	0.005	17.258	< .001***	0.117
EPEP 1 & 4	0.107	0.006	18.947	< .001***	0.147
EPEP 2 & 3	0.093	0.009	9.811	< .001***	0.119
LVE 1 & 2	0.110	0.006	17.849	< .001***	0.156
LVE 3 & 4	0.073	0.005	15.576	< .001***	0.123
IPPA 15 & 19	0.060	0.006	9.948	< .001***	0.069
IPPA 16 & 24	0.076	0.006	13.107	< .001***	0.090
IPPA 13 & 21	0.077	0.005	16.975	< .001***	0.104
IPPA 1 & 4	0.046	0.011	4.197	.001**	0.063

Parameters	Estimate
R^2 (Reliabilities)	
Composite[1]	
Parcels	
GSD	0.819
IWAH 1 & 4	0.636
IWAH 2 & 3	0.747
GSI	0.891
IWAH 6 & 7	0.797
IWAH 8 & 9	0.810
WrS	0.929
ZKE 1 & 3	0.852
ZKE 2 & 4	0.883
PoP	0.928
EPEP 1 & 4	0.853
EPEP 2 & 3	0.881
Lve	0.924
LVE 1 & 2	0.844
LVE 3 & 4	0.877
Cmm	0.950
IPPA 15 & 19	0.931
IPPA 16 & 24	0.910
Trs	0.956
IPPA 13 & 21	0.896
IPPA 1 & 4	0.937

Notes. N = 1539. *SE* = Standard error, Std. estimate = Standardized estimate (estimate in the fully standardized model). GSD = Global self-definition, GSI = Global self-investment, WrS = Warmth / Support, PoP = Positive parenting, Lve = Love, Trs = Trust, Cmm = Communication. The following abbreviations were used for the questionnaires: ZKE = „Zürcher Kurzfragebogen zum Erziehungsverhalten", EPEP = "Evaluation des Pratiques Educatives Parentales", LVE = items measuring parental love, IPPA = "Inventory of Parent and Peer Attachment", IWAH = "Identification With All Humanity scale". [1] Composite reliabilities of the factors (McDonald's ω) were calculated with the reliability() function of the R package "semTools" (Jorgensen et al., 2022). For factors with cross-loadings (PoP and Trs), reliabilities were calculated using formula (2) in Raykov and Shrout (2002). *p < .05. **p < .01. ***p < .001.

Table S8

Negative Model Allowing for all Correlations in the German Sample: Estimated Parameters

Parameters	Estimate	*SE*	*z*-value	*p*-value	Std. estimate
Factor loadings					
GSD → IWAH 1 & 4	1				0.859
GSD → IWAH 2 & 3	1.131	0.029	38.934	< .001***	0.892
GSI → IWAH 6 & 7	1				0.907
GSI → IWAH 8 & 9	1.026	0.024	43.234	< .001***	0.881
Ind → MOPS 5 & 13	1				0.874
Ind → MOPS 12 & 8	1.142	0.029	39.790	< .001***	0.912
Abs → MOPS 2 & 14	1				0.903
Abs → MOPS 15 & 7	1.051	0.024	43.195	< .001***	0.890
Ovr → MOPS 1 & 4	1				0.596
Ovr → MOPS 3 & 6	1.721	0.095	18.189	< .001***	0.866
Covariances					
GSD, GSI	2.193	0.103	21.308	< .001***	0.775
Ind	-0.016	0.031	-0.516	0.606	-0.017
Abs	0.028	0.031	0.892	0.372	0.030
Ovr	0.022	0.119	1.146	0.252	0.042
GSI, Ind	-0.054	0.037	-1.453	0.146	-0.046
Abs	0.021	0.035	0.585	0.558	0.017
Ovr	0.005	0.022	0.208	0.835	0.007
Ind, Abs	0.342	0.019	17.795	< .001***	0.891
Ovr	0.151	0.012	13.018	< .001***	0.712
Abs, Ovr	0.195	0.014	13.873	< .001***	0.905
Variances					
GSD	2.249	0.117	19.151	< .001***	1
GSI	3.561	0.155	22.973	< .001***	1
Ind	0.378	0.022	16.985	< .001***	1
Abs	0.391	0.023	17.316	< .001***	1
Ovr	0.119	0.012	9.629	< .001***	1
IWAH 1 & 4	0.798	0.060	13.328	< .001***	0.262
IWAH 2 & 3	0.740	0.070	10.621	< .001***	0.205
IWAH 6 & 7	0.766	0.076	10.067	< .001***	0.177

Parameters	Estimate	*SE*	*z*-value	*p*-value	Std. estimate
Variances					
IWAH 8 & 9	1.076	0.085	12.713	< .001***	0.223
MOPS 5 & 13	0.117	0.009	13.206	< .001***	0.236
MOPS 12 & 8	0.100	0.010	10.137	< .001***	0.168
MOPS 2 & 14	0.088	0.007	12.308	< .001***	0.184
MOPS 15 & 7	0.113	0.008	13.496	< .001***	0.207
MOPS 1 & 4	0.215	0.010	21.361	< .001***	0.644
MOPS 3 & 6	0.117	0.014	8.286	< .001***	0.249
R^2 (Reliabilities)					
Composite[1]					
Parcels					
GSD	0.869				
IWAH 1 & 4	0.738				
IWAH 2 & 3	0.795				
GSI	0.888				
IWAH 6 & 7	0.823				
IWAH 8 & 9	0.777				
Ind	0.889				
MOPS 5 & 13	0.764				
MOPS 12 & 8	0.832				
Abs	0.891				
MOPS 2 & 14	0.816				
MOPS 15 & 7	0.793				
Ovr	0.726				
MOPS 1 & 4	0.356				
MOPS 3 & 6	0.751				

Notes. *N* = 1517. *SE* = Standard error, Std. estimate = Standardized estimate (estimate in the fully standardized model). GSD = Global self-definition, GSI = Global self-investment, Ind = Indiffernce, Abs = Abuse, Ovr = Over-control. The following abbreviations were used for the two different questionnaires: MOPS = “Measure of Parental Style”, IWAH = “Identification With All Humanity scale”. [1] Composite reliabilities of the factors (McDonald’s ω) were calculated with the reliability() function of the R package “semTools” (Jorgensen et al., 2022). *p < .05. **p < .01. ***p < .001.

Table S9

Negative Model Allowing for all Correlations in the English Sample: Estimated Parameters

Parameters	Estimate	*SE*	*z*-value	*p*-value	Std. estimate
Factor loadings					
GSD → IWAH 1 & 4	1				0.790
GSD → IWAH 2 & 3	1.173	0.042	28.170	< .001***	0.873
GSI → IWAH 6 & 7	1				0.891
GSI → IWAH 8 & 9	1.042	0.027	38.017	< .001***	0.901
Ind → MOPS 5 & 13	1				0.943
Ind → MOPS 12 & 8	0.968	0.018	53.174	< .001***	0.964
Abs → MOPS 2 & 14	1				0.924
Abs → MOPS 15 & 7	1.052	0.024	43.709	< .001***	0.934
Ovr → MOPS 1 & 4	1				0.676
Ovr → MOPS 3 & 6	1.621	0.093	17.369	< .001***	0.938
Covariances					
GSD, GSI	1.870	0.096	19.514	< .001***	0.690
Ind	0.054	0.030	1.806	0.071	0.058
Abs	0.120	0.027	4.398	< .001***	0.153
Ovr	0.087	0.023	3.737	< .001***	0.151
GSI, Ind	-0.051	0.038	-1.357	0.175	-0.040
Abs	0.057	0.032	1.758	0.079	0.053
Ovr	0.070	0.027	2.546	0.011*	0.088
Ind, Abs	0.312	0.022	14.445	< .001***	0.844
Ovr	0.177	0.014	12.975	< .001***	0.648
Abs, Ovr	0.174	0.015	11.445	< .001***	0.761
Variances					
GSD	1.982	0.121	16.423	< .001***	1
GSI	3.701	0.173	21.355	< .001***	1
Ind	0.444	0.026	17.215	< .001***	1
Abs	0.309	0.024	12.968	< .001***	1
Ovr	0.168	0.016	10.419	< .001***	1
IWAH 1 & 4	1.195	0.076	15.753	< .001***	0.376
IWAH 2 & 3	0.851	0.096	8.879	< .001***	0.238
IWAH 6 & 7	0.956	0.097	9.830	< .001***	0.205

Parameters	Estimate	*SE*	*z*-value	*p*-value	Std. estimate
Variances					
IWAH 8 & 9	0.927	0.098	9.438	< .001***	0.187
MOPS 5 & 13	0.056	0.006	8.769	< .001***	0.112
MOPS 12 & 8	0.032	0.006	5.448	< .001***	0.070
MOPS 2 & 14	0.053	0.005	10.086	< .001***	0.146
MOPS 15 & 7	0.050	0.006	7.974	< .001***	0.127
MOPS 1 & 4	0.200	0.009	21.984	< .001***	0.543
MOPS 3 & 6	0.061	0.018	3.406	.001**	0.121
R^2 (Reliabilities)					
Composite[1]					
Parcels					
GSD	0.821				
IWAH 1 & 4	0.624				
IWAH 2 & 3	0.762				
GSI	0.891				
IWAH 6 & 7	0.795				
IWAH 8 & 9	0.813				
Ind	0.952				
MOPS 5 & 13	0.888				
MOPS 12 & 8	0.930				
Abs	0.927				
MOPS 2 & 14	0.854				
MOPS 15 & 7	0.873				
Ovr	0.816				
MOPS 1 & 4	0.457				
MOPS 3 & 6	0.879				

Notes. *N* = 1539. *SE* = Standard error, Std. estimate = Standardized estimate (estimate in the fully standardized model). GSD = Global self-definition, GSI = Global self-investment, Ind = Indiffernce, Abs = Abuse, Ovr = Over-control. The following abbreviations were used for the two different questionnaires: MOPS = “Measure of Parental Style”, IWAH = “Identification With All Humanity scale”. [1] Composite reliabilities of the factors (McDonald’s ω) were calculated with the reliability() function of the R package “semTools” (Jorgensen et al., 2022). *$p < .05$. **$p < .01$. ***$p < .001$.

Table S10

Estimated Parameters of the Positive Regression Model in the German Sample

Parameters	Estimate	*SE*	*z*-value	*p*-value	Std. estimate
Factor loadings					
GSD → IWAH 1 & 4	1				0.863
GSD → IWAH 2 & 3	1.121	0.029	39.130	< .001***	0.888
GSI → IWAH 6 & 7	1				0.913
GSI → IWAH 8 & 9	1.013	0.024	42.691	< .001***	0.876
WrS → ZKE 1 & 3	1				0.936
WrS → ZKE 2 & 4	1.003	0.013	80.107	< .001***	0.942
PoP → EPEP 1 & 4	1				0.669
PoP → EPEP 2 & 3	1.411	0.113	12.442	< .001***	0.912
Lve → LVE 1 & 2	1				0.946
Lve → LVE 3 & 4	0.962	0.011	88.501	< .001***	0.950
Cmm → IPPA 15 & 19	1				0.961
Cmm → IPPA 16 & 24	0.978	0.010	100.705	< .001***	0.953
Cmm → IPPA 13 & 21	0.514	0.028	18.096	< .001***	0.530
Cmm → EPEP 1 & 4	0.274	0.052	5.298	< .001***	0.288
Trs → IPPA 13 & 21	1				0.460
Trs → IPPA 1 & 4	2.154	0.145	14.889	< .001***	0.978
Regression coefficients					
GSD, WrS	0.952	1.030	0.924	0.356	0.506
PoP	0.101	0.679	0.149	0.881	0.039
Lve	-1.068	0.714	-1.495	0.135	-0.590
Trs	-0.091	0.437	-0.208	0.836	-0.024
Cmm	0.372	0.189	1.970	0.049*	0.223
GSI, WrS	0.359	1.204	0.298	0.766	0.151
PoP	1.038	0.792	1.311	0.190	0.313
Lve	-0.197	0.874	-0.226	0.822	-0.086
Trs	-0.746	0.528	-1.412	0.158	-0.158
Cmm	-0.167	0.241	-0.691	0.489	-0.079

Parameters	Estimate	*SE*	*z*-value	*p*-value	Std. estimate
Covariances					
GSD, GSI	2.126	0.103	20.636	< .001***	0.775
WrS, PoP	0.445	0.037	12.019	< .001***	0.970
Lve	0.664	0.021	31.486	< .001***	0.996
Cmm	0.632	0.021	30.702	< .001***	0.876
Trs	0.294	0.022	13.457	< .001***	0.912
PoP, Lve	0.435	0.036	12.059	< .001***	0.910
Cmm	0.469	0.041	11.387	< .001***	0.907
Trs	0.195	0.021	9.106	< .001***	0.845
Love, Cmm	0.635	0.021	30.575	< .001***	0.847
Trs	0.309	0.023	13.516	< .001***	0.921
Cmm, Trs	0.312	0.023	13.390	< .001***	0.859
Variances					
GSD	2.176	0.115	18.970	< .001***	0.959
GSI	3.457	0.155	22.319	< .001***	0.959
WrS	0.640	0.022	29.388	< .001***	1
PoP	0.329	0.052	6.320	< .001***	1
Lve	0.693	0.023	29.623	< .001***	1
Cmm	0.812	0.025	33.002	< .001***	1
Trs	0.162	0.021	7.775	< .001***	1
IWAH 1 & 4	0.778	0.059	13.237	< .001***	0.256
IWAH 2 & 3	0.765	0.068	11.227	< .001***	0.211
IWAH 6 & 7	0.721	0.075	9.574	< .001***	0.167
IWAH 8 & 9	1.123	0.086	13.088	< .001***	0.233
ZKE 1 & 3	0.090	0.005	17.304	< .001***	0.124
ZKE 2 & 4	0.081	0.005	16.812	< .001***	0.112
EPEP 1 & 4	0.088	0.005	16.854	< .001***	0.119
EPEP 2 & 3	0.132	0.010	12.902	< .001***	0.168
LVE 1 & 2	0.082	0.006	14.576	< .001***	0.106
LVE 3 & 4	0.069	0.007	10.085	< .001***	0.097
IPPA 15 & 19	0.067	0.006	11.335	< .001***	0.076
IPPA 16 & 24	0.079	0.006	13.559	< .001***	0.092
IPPA 13 & 21	0.068	0.004	15.903	< .001***	0.088
IPPA 1 & 4	0.034	0.011	3.221	.001**	0.044

Parameters	Estimate	*SE*	*z*-value	*p*-value	Std. estimate
R^2 (Coefficient of determination / Reliabilities)					
GSD	0.041				
GSI	0.041				
IWAH 1 & 4	0.744				
IWAH 2 & 3	0.789				
IWAH 6 & 7	0.833				
IWAH 8 & 9	0.767				
ZKE 1 & 3	0.876				
ZKE 2 & 4	0.888				
EPEP 1 & 4	0.881				
EPEP 2 & 3	0.832				
LVE 1 & 2	0.894				
LVE 3 & 4	0.903				
IPPA 15 & 19	0.924				
IPPA 16 & 24	0.908				
IPPA 13 & 21	0.912				
IPPA 1 & 4	0.956				

Notes. N = 1517. *SE* = Standard error, Std. estimate = Standardized estimate (estimate in the fully standardized model). GSD = Global self-definition, GSI = Global self-investment, WrS = Warmth / Support, PoP = Positive parenting, Lve = Love, Trs = Trust, Cmm = Communication. The following abbreviations were used for the questionnaires: ZKE = „Zürcher Kurzfragebogen zum Erziehungsverhalten", EPEP = "Evaluation des Pratiques Educatives Parentales", LVE = items measuring parental love, IPPA = "Inventory of Parent and Peer Attachment", IWAH = "Identification With All Humanity scale". *$p < .05$. **$p < .01$. ***$p < .001$.

Table S11

Estimated Parameters of the Positive Regression Model in the English Sample

Parameters	Estimate	*SE*	*z*-value	*p*-value	Std. estimate
Factor loadings					
GSD → IWAH 1 & 4	1				0.798
GSD → IWAH 2 & 3	1.150	0.041	27.789	< .001***	0.865
GSI → IWAH 6 & 7	1				0.893
GSI → IWAH 8 & 9	1.039	0.027	38.347	< .001***	0.900
WrS → ZKE 1 & 3	1				0.923
WrS → ZKE 2 & 4	1.012	0.013	79.899	< .001***	0.940
PoP → EPEP 1 & 4	1				0.651
PoP → EPEP 2 & 3	1.492	0.094	15.948	< .001***	0.938
Lve → LVE 1 & 2	1				0.919
Lve → LVE 3 & 4	0.928	0.014	64.525	< .001***	0.936
Cmm → IPPA 15 & 19	1				0.965
Cmm → IPPA 16 & 24	0.975	0.010	98.082	< .001***	0.954
Cmm → IPPA 13 & 21	0.493	0.030	16.327	< .001***	0.515
Cmm → EPEP 1 & 4	0.281	0.038	7.400	< .001***	0.297
Trs → IPPA 13 & 21	1				0.469
Trs → IPPA 1 & 4	2.044	0.147	13.937	< .001***	0.968
Regression coefficients					
GSD, WrS	-0.063	0.631	-0.099	0.921	-0.034
PoP	1.405	0.508	2.766	0.006**	0.547
Lve	-1.014	0.524	-1.937	0.053	-0.552
Trs	0.345	0.396	0.870	0.384	0.098
Cmm	0,179	0.167	1.071	0.284	0.113
GSI, WrS	-0.391	0.833	-0.469	0.639	-0.157
PoP	1.903	0.685	2.781	0.005**	0.547
Lve	-0.029	0.698	-0.041	0.967	-0.012
Trs	-0.153	0.537	-0.284	0.776	-0.032
Cmm	-0.399	0.220	-1.817	0.069	-0.186

Parameters	Estimate	*SE*	*z*-value	*p*-value	Std. estimate
Covariances					
GSD, GSI	1.787	0.097	18.388	< .001***	0.693
WrS, PoP	0.418	0.028	14.864	< .001***	0.971
Lve	0.608	0.021	28.904	< .001***	1.011
Cmm	0.616	0.020	30.745	< .001***	0.881
Trs	0.284	0.022	12.628	< .001***	0.904
PoP, Lve	0.400	0.027	14.886	< .001***	0.933
Cmm	0.439	0.031	14.309	< .001***	0.881
Trs	0.188	0.018	10.412	< .001***	0.838
Love, Cmm	0.587	0.020	29.534	< .001***	0.843
Trs	0.285	0.022	12.688	< .001***	0.911
Cmm, Trs	0.310	0.024	13.072	< .001***	0.851
Variances					
GSD	1.886	0.117	16.160	< .001***	0.933
GSI	3.524	0.168	20.962	< .001***	0.949
WrS	0.603	0.022	27.970	< .001***	1
PoP	0.618	0.021	29.912	< .001***	1
Lve	0.600	0.024	25.410	< .001***	1
Cmm	0.810	0.023	34.512	< .001***	1
Trs	0.164	0.022	7.376	< .001***	1
IWAH 1 & 4	1.156	0.076	15.143	< .001***	0.364
IWAH 2 & 3	0.904	0.097	9.307	< .001***	0.253
IWAH 6 & 7	0.944	0.097	9.782	< .001***	0.203
IWAH 8 & 9	0.940	0.097	9.730	< .001***	0.190
ZKE 1 & 3	0.105	0.006	18.450	< .001***	0.148
ZKE 2 & 4	0.082	0.005	17.258	< .001***	0.117
EPEP 1 & 4	0.107	0.006	18.947	< .001***	0.147
EPEP 2 & 3	0.093	0.009	9.811	< .001***	0.119
LVE 1 & 2	0.110	0.006	17.849	< .001***	0.156
LVE 3 & 4	0.073	0.005	15.576	< .001***	0.123
IPPA 15 & 19	0.060	0.006	9.948	< .001***	0.069
IPPA 16 & 24	0.076	0.006	13.107	< .001***	0.090
IPPA 13 & 21	0.077	0.005	16.975	< .001***	0.104
IPPA 1 & 4	0.046	0.011	4.197	< .001***	0.063

Parameters	Estimate	*SE*	*z*-value	*p*-value	Std. estimate
R^2 (Coefficient of determination / Reliabilities)					
GSD	0.067				
GSI	0.051				
IWAH 1 & 4	0.636				
IWAH 2 & 3	0.747				
IWAH 6 & 7	0.797				
IWAH 8 & 9	0.810				
ZKE 1 & 3	0.852				
ZKE 2 & 4	0.883				
EPEP 1 & 4	0.853				
EPEP 2 & 3	0.881				
LVE 1 & 2	0.844				
LVE 3 & 4	0.877				
IPPA 15 & 19	0.931				
IPPA 16 & 24	0.910				
IPPA 13 & 21	0.896				
IPPA 1 & 4	0.937				

Notes. *N* = 1539. *SE* = Standard error, Std. estimate = Standardized estimate (estimate in the fully standardized model). GSD = Global self-definition, GSI = Global self-investment, WrS = Warmth / Support, PoP = Positive parenting, Lve = Love, Trs = Trust, Cmm = Communication. The following abbreviations were used for the questionnaires: ZKE = „Zürcher Kurzfragebogen zum Erziehungsverhalten", EPEP = "Evaluation des Pratiques Educatives Parentales", LVE = items measuring parental love, IPPA = "Inventory of Parent and Peer Attachment", IWAH = "Identification With All Humanity scale". *p < .05. **p < .01. ***p < .001.

Table S12

Estimated Parameters of the Negative Regression Model in the English Sample

Parameters	Estimate	*SE*	*z*-value	*p*-value	Std. estimate
Factor loadings					
GSD → IWAH 1 & 4	1				0.790
GSD → IWAH 2 & 3	1.173	0.042	28.170	< .001***	0.873
GSI → IWAH 6 & 7	1				0.891
GSI → IWAH 8 & 9	1.042	0.027	38.017	< .001***	0.901
Ind → MOPS 5 & 13	1				0.943
Ind → MOPS 12 & 8	0.968	0.018	53.174	< .001***	0.964
Abs → MOPS 2 & 14	1				0.924
Abs → MOPS 15 & 7	1.052	0.024	43.709	< .001***	0.934
Ovr → MOPS 1 & 4	1				0.676
Ovr → MOPS 3 & 6	1.621	0.093	17.369	< .001***	0.938
Regression coefficients					
GSD, Ind	-0.526	0.138	-3.825	< .001***	-0.249
Abs	0.756	0.213	3.550	< .001***	0.298
Ovr	0.293	0.192	1.527	0.127	0.085
GSI, Ind	-0.854	0.178	-4.803	< .001***	-0.296
Abs	0.735	0.271	2.709	0.007**	0.212
Ovr	0.557	0.252	2.210	0.027*	0.119
Covariances					
GSD, GSI	1.779	0.092	19.415	< .001***	0.683
Ind, Abs	0.312	0.022	14.445	< .001***	0.844
Ovr	0.177	0.014	12.975	< .001***	0.648
Abs, Ovr	0.174	0.015	11.445	< .001***	0.761
Variances					
GSD	1.895	0.114	16.582	< .001***	0.956
GSI	3.577	0.167	21.408	< .001***	0.966
Ind	0.444	0.026	17.215	< .001***	1
Abs	0.309	0.024	12.968	< .001***	1
Ovr	0.168	0.016	10.419	< .001***	1
IWAH 1 & 4	1.195	0.076	15.753	< .001***	0.376
IWAH 2 & 3	0.851	0.096	8.879	< .001***	0.238

Parameters	Estimate	*SE*	*z*-value	*p*-value	Std. estimate
Variances					
IWAH 6 & 7	0.956	0.097	9.830	< .001***	0.205
IWAH 8 & 9	0.927	0.098	9.438	< .001***	0.187
MOPS 5 & 13	0.056	0.006	8.769	< .001***	0.112
MOPS 12 & 8	0.032	0.006	5.448	< .001***	0.070
MOPS 2 & 14	0.053	0.005	10.086	< .001***	0.146
MOPS 15 & 7	0.050	0.006	7.974	< .001***	0.127
MOPS 1 & 4	0.200	0.009	21.984	< .001***	0.543
MOPS 3 & 6	0.061	0.018	3.406	.001**	0.121
R^2 (Coefficient of determination / Reliabilities)					
GSD	0.044				
GSI	0.034				
IWAH 1 & 4	0.624				
IWAH 2 & 3	0.762				
IWAH 6 & 7	0.795				
IWAH 8 & 9	0.813				
MOPS 5 & 13	0.888				
MOPS 12 & 8	0.930				
MOPS 2 & 14	0.854				
MOPS 15 & 7	0.873				
MOPS 1 & 4	0.457				
MOPS 3 & 6	0.879				

Notes. *N* = 1539. *SE* = Standard error, Std. estimate = Standardized estimate (estimate in the fully standardized model). GSD = Global self-definition, GSI = Global self-investment, Ind = Indiffernce, Abs = Abuse, Ovr = Over-control. The following abbreviations were used for the two different questionnaires: MOPS = “Measure of Parental Style”, IWAH = “Identification With All Humanity scale”. **p* < .05. ***p* < .01. ****p* < .001.

Table S13

Estimated Parameters of the Combined Regression Model in the German Sample

Parameters	Estimate	*SE*	*z*-value	*p*-value	Std. estimate
Factor loadings					
GSD → IWAH 1 & 4	1				0.862
GSD → IWAH 2 & 3	1.122	0.029	39.100	< .001***	0.888
GSI → IWAH 6 & 7	1				0.912
GSI → IWAH 8 & 9	1.014	0.024	42.770	< .001***	0.876
WrS → ZKE 1 & 3	1				0.937
WrS → ZKE 2 & 4	1.002	0.013	79.914	< .001***	0.942
PoP → EPEP 1 & 4	1				0.674
PoP → EPEP 2 & 3	1.399	0.111	12.583	< .001***	0.912
Lve → LVE 1 & 2	1				0.946
Lve → LVE 3 & 4	0.962	0.011	88.539	< .001***	0.950
Cmm → IPPA 15 & 19	1				0.962
Cmm → IPPA 16 & 24	0.977	0.010	100.955	< .001***	0.952
Cmm → IPPA 13 & 21	0.496	0.026	18.759	< .001***	0.511
Cmm → EPEP 1 & 4	0.269	0.052	5.212	< .001***	0.183
Trs → IPPA 13 & 21	1				0.479
Trs → IPPA 1 & 4	2.057	0.120	17.083	< .001***	0.972
Ind → MOPS 5 & 13	1				0.870
Ind → MOPS 12 & 8	1.154	0.028	40.721	< .001***	0.917
Abs → MOPS 2 & 14	1				0.904
Abs → MOPS 15 & 7	1.049	0.024	43.773	< .001***	0.890
Ovr → MOPS 1 & 4	1				0.609
Ovr → MOPS 3 & 6	1.652	0.091	18.136	< .001***	0.849
Regression coefficients					
GSD, WrS	1.111	1.035	1.073	0.283	0.590
PoP	-0.164	0.685	-0.239	0.811	-0.063
Lve	-0.950	0.791	-1.201	0.230	-0.525
Trs	0.141	0.742	0.190	0.849	0.039
Cmm	0.370	0.218	1.699	0.089	0.222
Ind	-0.304	0.396	-0.768	0.443	-0.124
Abs	0.984	0.995	0.989	0.323	0.409
Ovr	-0.626	1.307	-0.479	0.632	-0.146

Parameters	Estimate	*SE*	*z*-value	*p*-value	Std. estimate
Regression coefficients					
GSI, WrS	0.873	1.353	0.646	0.518	0.368
PoP	0.527	0.915	0.576	0.565	0.160
Lve	0.311	1.072	0.290	0.772	0.136
Trs	-1.739	1.205	-1.444	0.149	-0.384
Cmm	0.038	0.325	0.116	0.908	0.018
Ind	-0.913	0.675	-1.351	0.177	-0.294
Abs	3.276	1.830	1.790	0.073	1.080
Ovr	-3.778	2.403	-1.572	0.116	-0.700
Covariances					
GSD, GSI	2.062	0.116	17.830	< .001***	0.780
WrS, PoP	0.448	0.037	12.152	< .001***	0.970
Lve	0.664	0.021	31.578	< .001***	0.996
Cmm	0.631	0.021	30.729	< .001***	0.875
Trs	0.307	0.020	15.062	< .001***	0.916
Ind	-0.362	0.018	-19.740	< .001***	-0.740
Abs	-0.324	0.018	-18.342	< .001***	-0.648
Ovr	-0.126	0.009	-13.681	< .001***	-0.450
PoP, Lve	0.438	0.036	12.193	< .001***	0.910
Cmm	0.472	0.041	11.492	< .001***	0.907
Trs	0.206	0.021	9.747	< .001***	0.851
Ind	-0.223	0.020	-11.144	< .001***	-0.633
Abs	-0.197	0.019	-10.484	< .001***	-0.544
Ovr	-0.071	0.008	-8.461	< .001***	-0.352
Love, Cmm	0.634	0.021	30.626	< .001***	0.845
Trs	0.323	0.021	15.179	< .001***	0.926
Ind	-0.396	0.019	-20.765	< .001***	-0.778
Abs	-0.360	0.019	-18.965	< .001***	-0.692
Ovr	-0.145	0.010	-14.808	< .001***	-0.495
Cmm, Trs	0.327	0.022	14.935	< .001***	0.864
Ind	-0.327	0.017	-18.988	< .001***	-0.593
Abs	-0.302	0.018	-17.160	< .001***	-0.536
Ovr	-0.119	0.010	-11.852	< .001***	-0.377

Parameters	Estimate	*SE*	*z*-value	*p*-value	Std. estimate
Covariances					
Trs, Ind	-0.188	0.014	-13.415	< .001***	-0.733
Abs	-0.186	0.014	-13.248	< .001***	-0.709
Ovr	-0.088	0.007	-12.262	< .001***	-0.597
Ind, Abs	0.340	0.019	17.881	< .001***	0.889
Ovr	0.154	0.012	13.240	< .001***	0.717
Abs, Ovr	0.202	0.014	14.238	< .001***	0.917
Variances					
GSD	2.137	0.115	18.640	< .001***	0.943
GSI	3.269	0.202	16.175	< .001***	0.908
WrS	0.640	0.022	29.414	< .001***	1.000
PoP	0.333	0.052	6.386	< .001***	1.000
Lve	0.693	0.023	29.707	< .001***	1.000
Cmm	0.812	0.025	33.083	< .001***	1.000
Trs	0.176	0.020	8.694	< .001***	1.000
Ind	0.375	0.022	17.070	< .001***	1.000
Abs	0.391	0.022	17.448	< .001***	1.000
Ovr	0.124	0.013	9.710	< .001***	1.000
IWAH 1 & 4	0.781	0.059	13.234	< .001***	0.256
IWAH 2 & 3	0.762	0.068	11.207	< .001***	0.211
IWAH 6 & 7	0.725	0.075	9.651	< .001***	0.168
IWAH 8 & 9	1.119	0.086	13.055	< .001***	0.232
ZKE 1 & 3	0.089	0.005	17.105	< .001***	0.123
ZKE 2 & 4	0.082	0.005	16.891	< .001***	0.113
EPEP 1 & 4	0.087	0.005	16.878	< .001***	0.119
EPEP 2 & 3	0.133	0.010	13.120	< .001***	0.169
LVE 1 & 2	0.081	0.006	14.315	< .001***	0.105
LVE 3 & 4	0.069	0.006	10.728	< .001***	0.097
IPPA 15 & 19	0.066	0.006	11.324	< .001***	0.075
IPPA 16 & 24	0.080	0.006	13.717	< .001***	0.093
IPPA 13 & 21	0.066	0.004	16.235	< .001***	0.086
IPPA 1 & 4	0.043	0.008	5.283	< .001***	0.055
MOPS 5 & 13	0.121	0.008	14.901	< .001***	0.244
MOPS 12 & 8	0.095	0.009	10.076	< .001***	0.160

Parameters	Estimate	*SE*	*z*-value	*p*-value	Std. estimate
MOPS 2 & 14	0.087	0.007	12.556	< .001***	0.183
MOPS 15 & 7	0.114	0.008	13.523	< .001***	0.208
MOPS 1 & 4	0.210	0.010	21.211	< .001***	0.629
MOPS 3 & 6	0.131	0.014	9.447	< .001***	0.279
R^2 (Coefficient of determination / Reliabilities)					
GSD	0.057				
GSI	0.092				
IWAH 1 & 4	0.744				
IWAH 2 & 3	0.789				
IWAH 6 & 7	0.832				
IWAH 8 & 9	0.768				
ZKE 1 & 3	0.877				
ZKE 2 & 4	0.887				
EPEP 1 & 4	0.881				
EPEP 2 & 3	0.831				
LVE 1 & 2	0.895				
LVE 3 & 4	0.903				
IPPA 15 & 19	0.925				
IPPA 16 & 24	0.907				
IPPA 13 & 21	0.914				
IPPA 1 & 4	0.945				
MOPS 5 & 13	0.756				
MOPS 12 & 8	0.840				
MOPS 2 & 14	0.817				
MOPS 15 & 7	0.792				
MOPS 1 & 4	0.371				
MOPS 3 & 6	0.721				

Notes. *N* = 1517. *SE* = Standard error, Std. estimate = Standardized estimate (estimate in the fully standardized model). GSD = Global self-definition, GSI = Global self-investment, WrS = Warmth / Support, PoP = Positive parenting, Lve = Love, Trs = Trust, Cmm = Communication, Ind = Indiffernce, Abs = Abuse, Ovr = Over-control. The following abbreviations were used for the questionnaires: ZKE = „Zürcher Kurzfragebogen zum Erziehungsverhalten", EPEP = "Evaluation des Pratiques Educatives Parentales", LVE = items measuring parental love, IPPA = "Inventory of Parent and Peer Attachment", IWAH = "Identification With All Humanity scale".
*p < .05. **p < .01. ***. p < .001.

Table S14

Estimated Parameters of the Combined Regression Model in the English Sample

Parameters	Estimate	*SE*	*z*-value	*p*-value	Std. estimate
Factor loadings					
GSD → IWAH 1 & 4	1				0.802
GSD → IWAH 2 & 3	1.138	0.041	27.994	< .001***	0.860
GSI → IWAH 6 & 7	1				0.893
GSI → IWAH 8 & 9	1.038	0.027	38.076	< .001***	0.900
WrS → ZKE 1 & 3	1				0.924
WrS → ZKE 2 & 4	1.011	0.013	79.891	< .001***	0.939
PoP → EPEP 1 & 4	1				0.665
PoP → EPEP 2 & 3	1.458	0.091	15.950	< .001***	0.936
Lve → LVE 1 & 2	1				0.921
Lve → LVE 3 & 4	0.924	0.014	65.924	< .001***	0.934
Cmm → IPPA 15 & 19	1				0.965
Cmm → IPPA 16 & 24	0.976	0.010	97.775	< .001***	0.954
Cmm → IPPA 13 & 21	0.483	0.027	17.994	< .001***	0.505
Cmm → EPEP 1 & 4	0.268	0.039	6.900	< .001***	0.283
Trs → IPPA 13 & 21	1				0.479
Trs → IPPA 1 & 4	1.995	0.121	16.519	< .001***	0.965
Ind → MOPS 5 & 13	1				0.948
Ind → MOPS 12 & 8	0.958	0.017	57.814	< .001***	0.959
Abs → MOPS 2 & 14	1				0.925
Abs → MOPS 15 & 7	1.049	0.024	43.888	< .001***	0.933
Ovr → MOPS 1 & 4	1				0.654
Ovr → MOPS 3 & 6	1.734	0.115	15.014	< .001***	0.969
Regression coefficients					
GSD, WrS	0.047	0.596	0.079	0.937	0.026
PoP	1.141	0.646	1.767	0.077	0.452
Lve	-1.056	0.561	-1.881	0.060	-0.574
Trs	1.000	0.476	2.102	0.036*	0.288
Cmm	0.084	0.166	0.504	0.614	0.053
Ind	-0.441	0.199	-2.220	0.026*	-0.207
Abs	0.588	0.212	2.774	0.006**	0.229
Ovr	0.623	0.228	2.735	0.006**	0.173

Parameters	Estimate	*SE*	*z*-value	*p*-value	Std. estimate
Regression coefficients					
GSI, WrS	-0.253	0.779	-0.325	0.745	-0.102
PoP	1.708	0.867	1.969	0.049	0.501
Lve	-0.155	0.748	0.208	0.835	-0.063
Trs	0.517	0.635	0.815	0.415	0.111
Cmm	-0.523	0.223	-2.345	0.019	-0.244
Ind	-0.554	0.271	-2.041	0.041	-0.193
Abs	0.746	0.272	2.746	0.006	0.215
Ovr	0.550	0.280	1.964	0.050	0.113
Covariances					
GSD, GSI	1.696	0.094	18.037	< .001***	0.682
WrS, PoP	0.428	0.029	14.924	< .001***	0.972
Lve	0.612	0.021	29.173	< .001***	1.012
Cmm	0.616	0.020	30.776	< .001***	0.880
Trs	0.291	0.020	14.522	< .001***	0.907
Ind	-0.330	0.020	-16.677	< .001***	-0.632
Abs	-0.218	0.017	-12.986	< .001***	-0.502
Ovr	-0.142	0.009	-15.509	< .001***	-0.458
PoP, Lve	0.411	0.027	14.961	< .001***	0.934
Cmm	0.449	0.031	14.303	< .001***	0.883
Trs	0.197	0.017	11.334	< .001***	0.842
Ind	-0.189	0.016	11.493	< .001***	-0.498
Abs	-0.121	0.013	-9.504	< .001***	-0.386
Ovr	-0.082	0.008	-10.508	< .001***	-0.364
Love, Cmm	0.588	0.020	29.631	< .001***	0.841
Trs	0.293	0.020	14.541	< .001***	0.913
Ind	-0.364	0.021	-17.170	< .001***	-0.699
Abs	-0.246	0.018	-13.612	< .001***	-0.568
Ovr	-0.155	0.009	-16.452	< .001***	-0.503
Cmm, Trs	0.317	0.021	15.007	< .001***	0.853
Ind	-0.272	0.019	14.424	< .001***	-0.451
Abs	-0.169	0.016	-10.534	< .001***	-0.339
Ovr	-0.140	0.010	-14.399	< .001***	-0.393

Parameters	Estimate	*SE*	*z*-value	*p*-value	Std. estimate
Covariances					
Trs, Ind	-0.168	0.014	-12.255	< .001***	-0.608
Abs	-0.118	0.011	-10.750	< .001***	-0.516
Ovr	-0.094	0.008	-12.260	< .001***	-0.577
Ind, Abs	0.315	0.022	14.547	< .001***	0.844
Ovr	0.170	0.014	11.959	< .001***	0.638
Abs, Ovr	0.163	0.016	10.351	< .001***	0.739
Variances					
GSD	1.810	0.111	16.253	< .001***	0.886
GSI	3.415	0.165	20.719	< .001***	0.919
WrS	0.606	0.022	28.064	< .001***	1.000
PoP	0.320	0.039	8.304	< .001***	1.000
Lve	0.604	0.024	25.719	< .001***	1.000
Cmm	0.808	0.023	34.482	< .001***	1.000
Trs	0.170	0.020	8.509	< .001***	1.000
Ind	0.450	0.026	17.444	< .001***	1.000
Abs	0.309	0.024	13.010	< .001***	1.000
Ovr	0.157	0.016	9.580	< .001***	1.000
IWAH 1 & 4	1.133	0.076	14.980	< .001***	0.357
IWAH 2 & 3	0.934	0.095	9.846	< .001***	0.261
IWAH 6 & 7	0.941	0.097	9.684	< .001***	0.202
IWAH 8 & 9	0.943	0.097	9.691	< .001***	0.191
ZKE 1 & 3	0.104	0.006	18.296	< .001***	0.147
ZKE 2 & 4	0.083	0.005	17.338	< .001***	0.118
EPEP 1 & 4	0.105	0.006	18.666	< .001***	0.145
EPEP 2 & 3	0.096	0.009	10.475	< .001***	0.124
LVE 1 & 2	0.108	0.006	17.860	< .001***	0.151
LVE 3 & 4	0.075	0.005	16.499	< .001***	0.127
IPPA 15 & 19	0.060	0.006	9.995	< .001***	0.070
IPPA 16 & 24	0.076	0.006	12.974	< .001***	0.090
IPPA 13 & 21	0.076	0.004	17.453	< .001***	0.102
IPPA 1 & 4	0.051	0.009	5.803	< .001***	0.069
MOPS 5 & 13	0.051	0.006	9.096	< .001***	0.101
MOPS 12 & 8	0.036	0.005	7.133	< .001***	0.081

Parameters	Estimate	*SE*	*z*-value	*p*-value	Std. estimate
MOPS 2 & 14	0.052	0.005	10.053	< .001***	0.144
MOPS 15 & 7	0.051	0.006	8.184	< .001***	0.129
MOPS 1 & 4	0.210	0.010	20.570	< .001***	0.572
MOPS 3 & 6	0.031	0.022	1.369	< .001***	0.061
R^2 (Coefficient of determination / Reliabilities)					
GSD	0.114				
GSI	0.081				
IWAH 1 & 4	0.643				
IWAH 2 & 3	0.739				
IWAH 6 & 7	0.798				
IWAH 8 & 9	0.809				
ZKE 1 & 3	0.853				
ZKE 2 & 4	0.882				
EPEP 1 & 4	0.855				
EPEP 2 & 3	0.876				
LVE 1 & 2	0.849				
LVE 3 & 4	0.873				
IPPA 15 & 19	0.930				
IPPA 16 & 24	0.910				
IPPA 13 & 21	0.898				
IPPA 1 & 4	0.931				
MOPS 5 & 13	0.899				
MOPS 12 & 8	0.919				
MOPS 2 & 14	0.856				
MOPS 15 & 7	0.871				
MOPS 1 & 4	0.428				
MOPS 3 & 6	0.939				

Notes. *N* = 1539. *SE* = Standard error, Std. estimate = Standardized estimate (estimate in the fully standardized model). GSD = Global self-definition, GSI = Global self-investment, WrS = Warmth / Support, PoP = Positive parenting, Lve = Love, Trs = Trust, Cmm = Communication, Ind = Indiffernce, Abs = Abuse, Ovr = Over-control. The following abbreviations were used for the questionnaires: ZKE = „Zürcher Kurzfragebogen zum Erziehungsverhalten”, EPEP = “Evaluation des Pratiques Educatives Parentales”, LVE = items measuring parental love, IPPA = “Inventory of Parent and Peer Attachment”, IWAH = “Identification With All Humanity scale”.
p* < .05. ***p* < .01. *. *p* < .001.

2 Figures

Figure S1. Density Plots for Global Self-Definition (GSD) and Global Self-Investment (GSI)

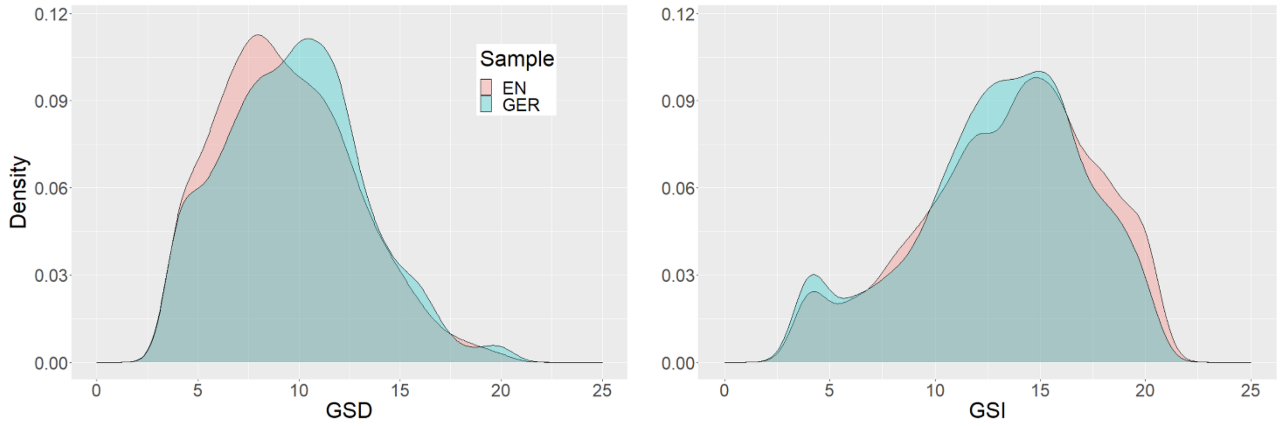

Notes. EN = English sample (red), GER = German sample (blue). Scales for GSD and GSI range from 4 to 20.

Figure S2. Density Plots for pRPB Variables: Warmth/Support, Positive Parenting, and Love

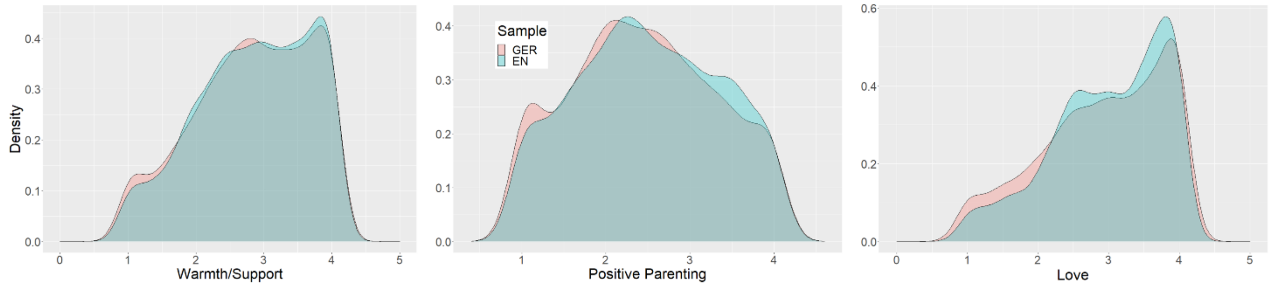

Notes. pRPB = Positive remembered parenting behavior. EN = English sample (red), GER = German sample (blue). Scales for all variables range from 1 to 4 as they were calculated by averaging the scores over all items belonging to one variable for both parents.

Figure S3. Density Plots for nRPB Variables: Indifference, Abuse, and Over-Control

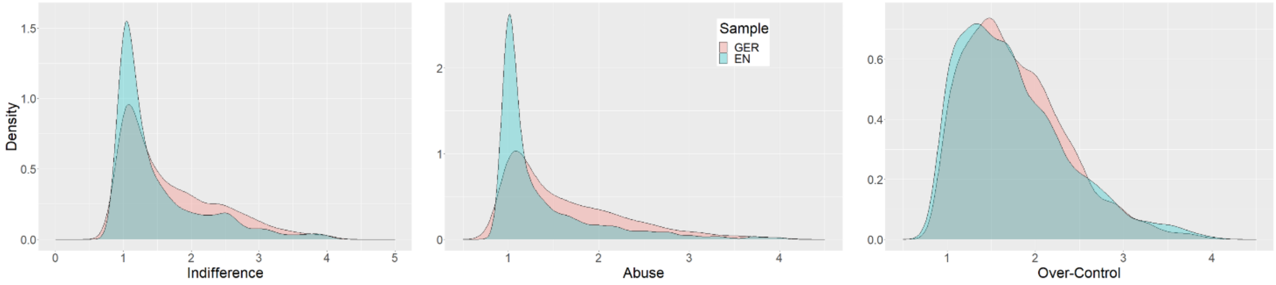

Notes. nRPB = Negative remembered parenting behavior. EN = English sample (red), GER = German sample (blue). All 15 items from the Measure of Parental Style (MOPS) were assessed by the participants. Scales for all variables range from 1 to 4 as they were calculated by averaging the scores over all items belonging to one variable for both parents.

Figure S4. Density Plots for Attachment Variables: Communication and Trust

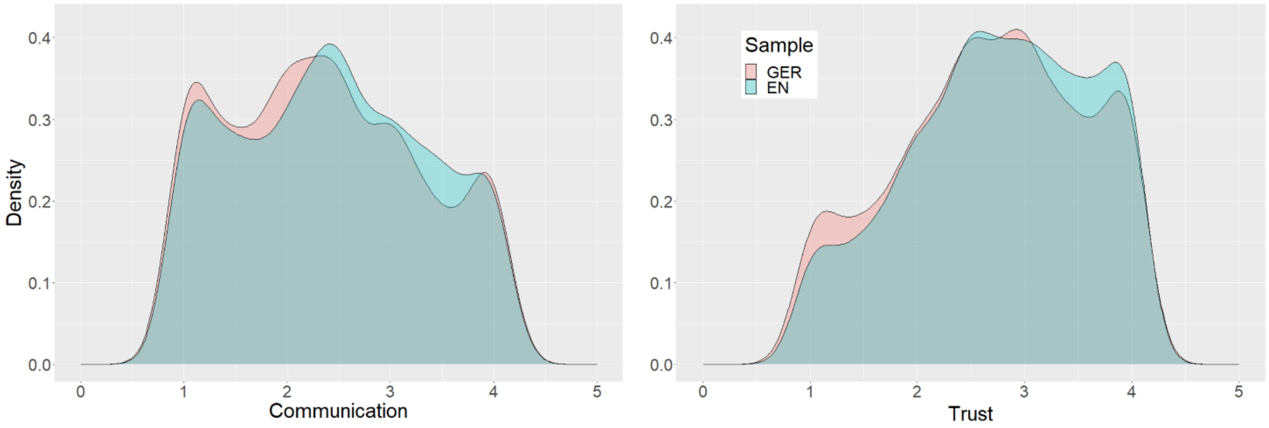

Notes. EN = English sample (red), GER = German sample (blue). Scales for all variables range from 1 to 4 as they were calculated by averaging the scores over all items belonging to one variable for both parents.

3 Identification With All Humanity Scale

The English and the German version of the IWAH scale as used in the present study:

Item	**English version**, adapted version of the original scale by McFarland et al. (2012)	**German version**, adapted version of the German scale by Reese et al. (2015)
IWAH1	How close do you feel to each of the following groups? Please choose the letter that best represents your feelings on the following scale:<br><br>A = not at all close; B = not very close; C = just a little or somewhat close; D = pretty close; E = very close<br><br>1. People in my community<br>2. English people<br>3. Europeans<br>4. Adherents of my religion / denomination[1]<br>5. People all over the world	Wie nahe fühlen Sie sich jeder dieser folgenden Gruppen? Bitte wählen Sie den Buchstaben aus, der auf der folgenden Skala Ihre Gefühle am besten wiedergibt:<br><br>A = gar nicht nahe; B = nicht sehr nahe; C = ein bisschen nahe; D = ziemlich nahe;<br>E = sehr nahe<br><br>1. Menschen in meinem persönlichen Umfeld<br>2. Deutsche<br>3. Europäer*innen<br>4. Anhänger*innen meiner Religion / Konfession[1]<br>5. Menschen auf der ganzen Welt
IWAH2	How often do you use the word “we” to refer to the following groups of people?<br><br>A = almost never; B = rarely; C = occasionally; D = often; E = very often<br><br>1. People in my community<br>2. English people<br>3. Europeans<br>4. Adherents of my religion / denomination[1]<br>5. People all over the world	Wie oft benutzen Sie das Wort „wir“, wenn Sie über die folgenden Gruppen sprechen?<br><br>A = fast nie; B = selten; C = ab und zu; D = oft; E = sehr oft<br><br>1. Menschen in meinem persönlichen Umfeld<br>2. Deutsche<br>3. Europäer*innen<br>4. Anhänger*innen meiner Religion/Konfession[1]<br>5. Menschen auf der ganzen Welt

IWAH3	How much would you say you have in common with the following groups?<br><br>A = almost nothing in common; B = little in common; C = some in common; D = quite a bit in common; E = very much in common<br><br>1. People in my community<br>2. English people<br>3. Europeans<br>4. Adherents of my religion / denomination[1]<br>5. People all over the world	Wie viel haben Sie Ihrer Meinung nach mit den folgenden Gruppen gemeinsam?<br><br>A = fast nichts gemeinsam; B = wenig gemeinsam; C = etwas gemeinsam; D = ziemlich viel gemeinsam; E = sehr viel gemeinsam<br><br>1. Menschen in meinem persönlichen Umfeld<br>2. Deutsche<br>3. Europäer*innen<br>4. Anhänger*innen meiner Religion/Konfession[1]<br>5. Menschen auf der ganzen Welt
IWAH4	Sometimes people think of those who are not part of their immediate family as “family”. To what degree do you think of the following groups of people as “family”?<br><br>1. People in my community<br>2. English people<br>3. Europeans<br>4. Adherents of my religion / denomination[1]<br>5. All humans everywhere[2]<br><br>A = not at all; B = just a little; C = somewhat; D = quite a bit; E = very much	Manchmal bezeichnen Menschen auf jene als „Familie“, die eigentlich nicht Teil der eigenen Familie sind. Wie sehr betrachten Sie die folgenden Gruppen als „Familie“?<br><br>1. Menschen in meinem persönlichen Umfeld<br>2. Deutsche<br>3. Europäer*innen<br>4. Anhänger*innen meiner Religion/Konfession[1]<br>5. Menschen auf der ganzen Welt<br><br>A = gar nicht; B = kaum; C = ein wenig; D = ziemlich; E = sehr
IWAH5	How much do you identify with (that is, feel a part of, feel love towards, have concern for) each of the following?<br><br>1. People in my community<br>2. English people<br>3. Europeans<br>4. Adherents of my religion / denomination[1]<br>5. All humans everywhere[2]<br><br>A = not at all; B = just a little; C = somewhat; D = quite a bit; E = very much	Wie sehr identifizieren Sie sich (d.h., fühlen Sie sich zugehörig, empfinden Sie Zuneigung, sorgen Sie sich um) mit jeder der folgenden Gruppen?<br><br>1. Menschen in meinem persönlichen Umfeld<br>2. Deutsche<br>3. Europäer*innen<br>4. Anhänger*innen meiner Religion/Konfession[1]<br>5. Menschen auf der ganzen Welt<br><br>A = gar nicht; B = kaum; C = ein wenig; D = ziemlich; E = sehr

IWAH6	How much would you say you care (feel upset, want to help) when bad things happen to:<br><br>1. People in my community<br>2. English people<br>3. Europeans<br>4. Adherents of my religion / denomination[1]<br>5. People anywhere in the world[2]<br><br>A = not at all; B = just a little; C = somewhat; D = quite a bit; E = very much	Wie betroffen fühlen Sie sich, wenn einer der folgenden Gruppen etwas Schlimmes passiert?<br><br>1. Menschen in meinem persönlichen Umfeld<br>2. Deutsche<br>3. Europäer*innen<br>4. Anhänger*innen meiner Religion/Konfession[1]<br>5. Menschen auf der ganzen Welt<br><br>A = gar nicht; B = kaum; C = ein wenig; D = ziemlich; E = sehr
IWAH7	How much do you want to be:<br><br>1. a responsible citizen of my community?<br>2. a responsible English citizen?<br>3. a responsible European citizen?<br>4. a responsible member of my religious community / denomination[1]<br>5. a responsible citizen of the world?<br><br>A = not at all; B = just a little; C = somewhat; D = quite a bit; E = very much	Wie sehr möchten Sie folgendes sein:<br><br>1. ein verantwortungsvolles Mitglied in meinem persönlichen Umfeld<br>2. ein*e verantwortungsvolle*r Bürger*in Deutschlands<br>3. ein*e verantwortungsvolle*r Bürger*in Europas<br>4. ein verantwortungsvolles Mitglied meiner Religion/Konfession[1]<br>5. ein*e verantwortungsvolle*r Bürger*in der Welt<br><br>A = gar nicht; B = kaum; C = ein wenig; D = ziemlich; E = sehr
IWAH8	How much do you believe in:<br><br>1. being loyal to your community?<br>2. being loyal to England?<br>3. being loyal to Europe?<br>4. being loyal to your religion / denomination? [1]<br>5. being loyal to all mankind?<br><br>A = not at all; B = just a little; C = somewhat; D = quite a bit; E = very much	Wie wichtig ist es Ihnen,<br><br>1. Ihrem persönlichen Umfeld gegenüber aufrichtig zu sein?<br>2. gegenüber Deutschland aufrichtig zu sein?<br>3. gegenüber Europa aufrichtig zu sein?<br>4. Ihrer Religion/Konfession gegenüber aufrichtig zu sein? [1]<br>5. gegenüber Menschen auf der ganzen Welt aufrichtig zu sein?<br><br>A = gar nicht; B = kaum; C = ein wenig; D = ziemlich; E = sehr

IWAH9	When they are in need, how much do you want to help:<br><br>1. People in my community?<br>2. English people?<br>3. Europeans?<br>4. Adherents of my religion / denomination[1]<br>5. People all over the world?<br><br>A = not at all; B = just a little; C = somewhat; D = quite a bit; E = very much	Wie sehr möchten Sie folgenden Gruppen helfen, wenn diese Hilfe benötigen?<br><br>1. Menschen in meinem persönlichen Umfeld Deutsche<br>2. Europäer*innen<br>3. Anhänger*innen meiner Religion/Konfession[1]<br>4. Menschen auf der ganzen Welt<br><br>A = gar nicht; B = kaum; C = ein wenig; D = ziemlich; E = sehr

Notes. [1] Option 4 (‘Adherents of my religion / denomination’) was only presented to participants who had reported to be members of a religious congregation or community. [2] Within the items 4 to 6 and in the English sample only, participants who had reported to be members of a religious congregation or community were accidentally presented a different fifth response option than the other, non-religious participants. Instead of the original (correct) names for the fifth response option displayed above, which were “All humans everywhere” (items 4 and 5) and „People anywhere in the world“ (item 6), religious participants were shown the option “People all over the world” for all three items.

4 References

Jorgensen, T. D., Pornprasertmanit, S., Schoemann, A. M., and Rosseel, Y. (2022). *semTools: Useful tools for structural equation modeling*. R package version 0.5-6. https://CRAN.R-project.org/package=semTools.

McFarland, S., Webb, M., and Brown, D. (2012). All humanity is my ingroup: a measure and studies of identification with all humanity. *Journal of Personality and Social Psychology* 103, 830–853. doi: 10.1037/a0028724.

Raykov, T., and Shrout, P. E. (2002). Reliability of scales with general structure: Point and interval estimation using a structural equation modeling approach. *Structural Equation Modeling: A Multidisciplinary Journal* 9, 195–212. doi: 10.1207/S15328007SEM0902_3.

Reese, G., Proch, J., and Finn, C. (2015). Identification with all humanity: The role of self-definition and self-investment. *European Journal of Social Psychology* 45, 426–440. doi: 10.1002/ejsp.2102.
